# Cationic antimicrobial peptide NRC-03 induces oral squamous cell carcinoma cell apoptosis via CypD-mPTP axis-mediated mitochondrial oxidative stress

**DOI:** 10.1016/j.redox.2022.102355

**Published:** 2022-05-28

**Authors:** Dan Hou, Fengjun Hu, Yixin Mao, Liang Yan, Yuhui Zhang, Zhichao Zheng, Antong Wu, Tymour Forouzanfar, Janak L. Pathak, Gang Wu

**Affiliations:** a Affiliated Stomatology Hospital of Guangzhou Medical University, Guangdong Engineering Research Center of Oral Restoration and Reconstruction, Guangzhou Key Laboratory of Basic and Applied Research of Oral Regenerative Medicine, Guangzhou, Guangdong, 510182, China; b Department of Oral and Maxillofacial Surgery/Oral Pathology, Amsterdam UMC/VUmc and Academic Centre for Dentistry Amsterdam (ACTA), Vrije Universiteit Amsterdam, Amsterdam Movement Science, Amsterdam, 1081 HZ, the Netherlands; c Institute of Information Technology, Zhejiang Shuren University, Hangzhou, Zhejiang, 310000, China; d Department of Prosthodontics, School and Hospital of Stomatology, Wenzhou Medical University, Wenzhou, 325027, China; e Institute of Stomatology, School and Hospital of Stomatology, Wenzhou Medical University, Wenzhou, 325027, China; f Laboratory for Myology, Department of Human Movement Sciences, Faculty of Behavioural and Movement Sciences, Vrije Universiteit Amsterdam, Amsterdam Movement Sciences, Amsterdam, 1081 HZ, Netherlands; g Department of Medical Biochemistry and Molecular Biology, School of Medicine, Jinan University, Guangzhou, 510632, China; h Department of Oral Cell Biology, Academic Centre of Dentistry Amsterdam (ACTA), University van Amsterdam and Vrije Universiteit Amsterdam, Amsterdam, 1081LA, Netherlands

**Keywords:** NRC-03, Oral squamous cell carcinoma, Cyclophilin D, Cell apoptosis, Oxidative stress

## Abstract

Pleurocidin-family cationic antimicrobial peptide NRC-03 exhibits potent and selective cytotoxicity towards cancer cells. However, the anticancer effect of NRC-03 in oral squamous cell carcinoma (OSCC) and the molecular mechanism of NRC-03 induced cancer cell death is still unclear. This study focused to investigate mitochondrial oxidative stress-mediated altered mitochondrial function involved in NRC-03-induced apoptosis of OSCC cells. NRC-03 entered the OSCC cells more easily than that of normal cells and bound to mitochondria as well as the nucleus, causing cell membrane blebbing, mitochondria swelling, and DNA fragmentation. NRC-03 induced high oxygen consumption, reactive oxygen species (ROS) release, mitochondrial dysfunction, and apoptosis in OSCC cells. Non-specific antioxidant *N*-acetyl-l-cysteine (NAC), or mitochondria-specific antioxidant mitoquinone (MitoQ) alleviated NRC-03-induced apoptosis and mitochondrial dysfunction indicated that NRC-03 exerts a cytotoxic effect in cancer cells via inducing cellular and mitochondrial oxidative stress. Moreover, the expression of cyclophilin D (CypD), the key component of mitochondrial permeability transition pore (mPTP), was upregulated in NRC-03-treated cancer cells. Blockade of CypD by siRNA-mediated depletion or pharmacological inhibitor cyclosporine A (CsA) significantly suppressed NRC-03-induced mitochondrial oxidative stress, mitochondrial dysfunction, and apoptosis. NRC-03 also activated MAPK/ERK and NF-κB pathways. Importantly, intratumoral administration of NRC-03 inhibited the growth of CAL-27 cells-derived tumors on xenografted animal models. Taken together, our study indicates that NRC-03 induces apoptosis in OSCC cells via the CypD-mPTP axis mediated mitochondrial oxidative stress.

## Highlights

• NRC-03 induces OSCC cell apoptosis and inhibits OSCC ectopic tumor growth in mice.
• NRC-03 binds to the plasma membrane via electrostatic interaction and enters into cells.
• NRC-03 binds to the mitochondrial membrane, enters the nucleus, induces mtROS.
• NRC-03 upregulates CypD expression that triggers mPTP opening and caspase cascade.
• NRC-03 causes mitochondrial dysfunction and DNA fragmentation to induce apoptosis.

## Graphical abstract

**Figure:**
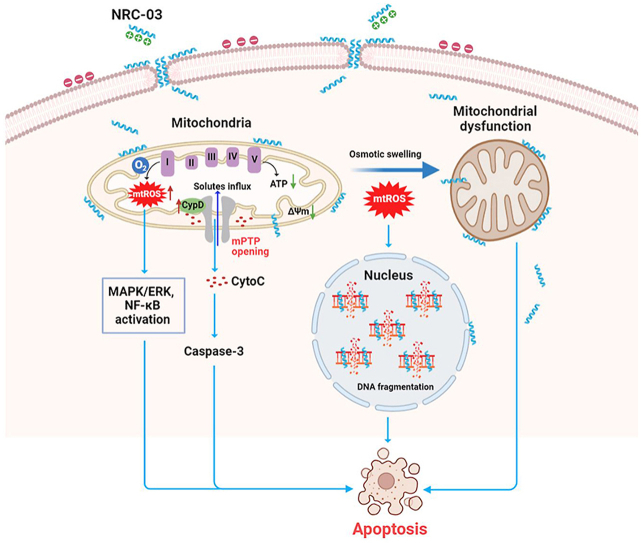

## 1 Introduction

Oral squamous cell carcinoma (OSCC) is the most common oral malignancy and represents around 90% of all cancers in the oral cavity [1], causing approximately 300,000 cases and 145,000 deaths worldwide annually [2,3]. OSCC in the initial stages shows an asymptomatic erytholeukoplastic lesion mainly on the tongue, mouth floor, buccal mucosa, gingiva, and lips [4]. In advanced stages of OSCC, the lesions may develop into ulcers and lumps with irregular and poorly defined margins, causing a series of severe symptoms, such as severe pain, bleeding, problems in breathing, and difficulty in speech [[5], [6], [7]]. Because early carcinomas are asymptomatic, most OSCC cases are usually diagnosed in advanced stages [8]. Moreover, OSCC exhibits high aggression, rapid progression, and early relapse, thus causing high mortality with a five-year survival rate of less than 50% [9]. Chemotherapy is a critical treatment option in a multimodality therapeutic approach to treat locally advanced tumors, nonresectable tumors, metastatic tumors, and palliative chemotherapy [[10], [11], [12]]. Cisplatin, carboplatin, 5-fluorouracil, paclitaxel, and docetaxel are the commonly used first-line chemotherapeutic drugs to treat OSCC with a mechanism of damaging cell replication machinery of rapidly dividing cells [6,13]. However, apart from cancer cells, these drugs also damage the healthy cells with a high division rate from bone marrow, hair follicles, and gastrointestinal tract, which leads to a series of severe side effects, such as myelosuppression, alopecia, rashes, and vomiting [14,15]. To approach this problem, continuous efforts have been taken to develop cancer-targeting chemotherapeutic drugs [16].

NRC-03, a 26-residue pleurocidin-like cationic antimicrobial peptides (CAPs), is derived from skin mucous secretions of winter flounder, and has promising cancer-targeting potential [17]. The cationic property allows NRC-03 to specifically target negatively charged cancer cells, thereby causing membrane disturbance and cell death. Recent studies have shown that NRC-03 selectively kills human breast cancer, multiple myeloma, and leukemia cells, but is less toxic to normal cells, such as human umbilical vein endothelial cells, dermal fibroblasts, and erythrocytes [[18], [19], [20], [21]]. However, the anticancer potential of NRC-03 towards OSCC has not been investigated yet. Furthermore, the mode and molecular mechanisms of NRC-03-induced cancer cell death remain largely unknown. One major subcellular target of CAPs is mitochondria which play an important role in tumor initiation and progression through ATP production, catabolic and anabolic metabolism, generation of ROS, and apoptosis [22]. Certain CAPs can interact with mitochondria directly or indirectly to trigger ROS generation, causing mitochondrial dysfunction and cancer cell death [[23], [24], [25], [26]]. In breast cancer cells, NRC-03 has been shown to co-localize with mitochondria, decrease mitochondrial membrane potential and cause the release of cytochrome *c* [18]. However, the underlying molecular mechanisms remain unclear. mPTP is a reversible mitochondrial channel, which plays an important physiological role in maintaining mitochondrial homeostasis by the timely discharge of ROS and Ca^2+^ from mitochondria [27]. In pathological conditions, such as oxidative stress, continuous mPTP openings cause a burst release of ROS, resulting in mitochondrial dysfunction [28,29], which forms a vicious circle leading to cell death [30]. CypD is a critical regulator for mPTP opening [31]. CypD-dependent mPTP opening has been shown to play a key role in ROS-induced mitochondrial dysfunction and cell death [31,32]. However, the role of CypD-dependent mitochondrial dysfunction in NRC-03-induced cancer cell death is still unclear.

In this study, we aimed to investigate the anti-OSCC efficacy of NRC-03 and its underlying mechanisms. We first assessed the cytotoxicity, apoptosis, and DNA damage potential of NRC-03 in two OSCC cell lines (CAL-27 and SCC-9) and normal human oral keratinocytes (HOK). We further investigated the OSCC growth inhibition potential of NRC-03 in nude mice ectopic tumor model. Thereafter, we monitored the interaction between NRC-03 and OSCC cells and evaluated the role of mitochondrial dysfunctions in NRC-03-induced apoptosis of OSCC cells. RNA sequencing was adopted to sort out the involved signaling pathways. Finally, we proved the critical role of CypD-mPTP openings in the anti-OSCC effect of NRC-03.

## 2 Materials and methods

### 2.1 Peptide synthesis

NRC-03 (GRRKRKWLRRIGKGVKIIGGAALDHL-NH_2_) and tetramethylrhodamine (TRITC)-labeled NRC-03 were synthesized by Top-peptide Co., Ltd. (Shanghai, China) via Fmoc solid-phase peptide synthesis. The purity of the peptide was over 95%. Molecular weight (MW): 2953.4, net charge: +9.5, and isoelectric point (p*I*):12.67 were the basic biochemical properties of NRC-03 ^19^. Lyophilized peptides were reconstituted in serum-free Dulbecco's Modified Eagle Medium/Nutrient Mixture F-12 (DMEM/F-12). All experiments were conducted in a medium containing 2.5% FBS to limit peptide degradation by serum proteases.

### 2.2 Reagents

Cell culture medium and supplements were purchased from GIBCO BRL (Gaithersburg, MD, USA). Anti-β-actin antibody (#4970) and anti-rabbit secondary antibody (#7074) were obtained from Cell Signaling Technology (MA, USA). Anti-CypD antibody (ab110324) and Hydrogen peroxide assay kit - (Fluorometric-Near Infrared) (ab138886) were from Abcam (MA, USA). Anti-mouse secondary antibody (sc-516102) was purchased from Santa Cruz Biotechnology, Inc. (Santa Cruz, CA). MitoSOX Red Mitochondrial Superoxide Indicator, MitoTracker Green FM, and LysoTracker Green DND-26 were from Yeasen biotechnologies Co., Ltd. (Shanghai, China). Terminal deoxynucleotidyl transferase dUTP nick-end labeling (TUNEL) kit, Caspase-3 activity assay kit, Caspase-8 activity assay kit, Bradford protein assay kit, Adenosine triphosphate (ATP) assay kit, Hoechst 33342 staining kit, NAC, phenylmethanesulfonyl fluoride (PMSF), and RIPA buffer were from Beyotime Institute of Biotechnology (Shanghai, China). Z-VAD-FMK and cyclosporine A (CsA) were purchased from MedChemExpress (Princeton, NJ, United States). Mitoquinone (MitoQ) was from Cayman Chemical Company (Ann Arbor, MI, USA). Oxygen consumption rate (OCR) assay kit, 2′,7′-dichlorodihydrofluorescein diacetate (DCFH-DA), annexin V-FITC/propidium iodide (Annexin V-PI) staining kit, mitochondrial membrane potential assay kit with JC-1, and BCA protein assay kit were purchased from BestBio biotechnologies Co., Ltd. (Shanghai, China). Lipofectamine 3000 transfection reagents were from Invitrogen (Carlsbad, CA, USA).

### 2.3 Cell lines and cell culture

OSCC cell lines CAL-27 and SCC-9 were purchased from ATCC and cultured in DMEM/F-12. HOK were obtained from ScienCell and cultured in Oral Keratinocyte Medium. The medium was supplemented with 10% fetal bovine serum (FBS) and 1% penicillin-streptomycin in 5% CO_2_ at 37 °C in a humidified incubator.

### 2.4 Cell treatment

Test compounds were prepared as stock solutions and diluted to the desired final concentrations immediately before use. The final concentrations of the compounds were as follows: NRC-03 (45 μg/ml), Z-VAD-FMK (50 μM), NAC (5 mM), MitoQ (1 μM), and CsA (2 μM). Cells were treated with or without NRC-03 and the indicated test compounds according to the experiment protocol.

### 2.5 Cell viability assay

In vitro cytotoxicity was determined by the Cell Counting Kit-8 (Dojindo Corp.) assay. Cells were plated in 96-well plates (1 × 10^4^ cells/well) and exposed to NRC-03 in the absence or presence of other test compounds. The cells were subsequently incubated for 3 h at 37 °C and the absorbance was measured at 450 nm using Multiskan™ FC Microplate Photometer (Thermo Scientific).

### 2.6 Colocalization analysis

A confocal laser scanning microscopy (CLSM, Leica TCS SP8) was performed to observe the subcellular localization of NRC-03. CAL-27 cells were seeded confocal dishes at a density of 2 × 10^5^ cells/well. After incubation for 24 h, the cells were treated with TRITC-labeled NRC-03 at predetermined intervals, cells were washed twice with cold PBS and then incubated with Mitotracker green (excitation: 488 nm, emission: 530 nm), LysoTracker Green (excitation: 504 nm, emission: 511 nm) or Hoechst 33342 (excitation: 350 nm, emission: 405 nm) according to the manufacturer's instructions, respectively.

### 2.7 Transmission electron microscope (TEM) analysis

CAL-27 cells were fixed overnight in 2.5% glutaraldehyde in phosphate buffer (0.1 M, pH7.0). After post-fixation with OsO_4_ in phosphate buffer (0.1 M, pH7.0), cells were dehydrated with a graded series of ethanol and embedded in resin. Ultra-thin (70–90 nm) sections were cut, stained by uranyl acetate and alkaline lead citrate, and observed in TEM (Hitachi H-7650).

### 2.8 Small interfering RNA (siRNA) transfection

CypD siRNA targeting human peptidylprolyl isomerase F (PPIF) and negative control (NC) were transfected with Lipofectamine 3000 in CAL-27 cells according to the manufacturer's instructions. The sequence of siRNA-CypD is GACGAGAACTTTACACTGA.

### 2.9 Measurement of apoptosis by flow cytometry and TUNEL assay

CAL-27 cells were cultured in 60-mm dishes and treated with or without other test compounds for 1 h prior to NRC-03 treatment for 4 h, then trypsinized, washed with PBS, and centrifuged at 1000 rpm for 5 min. Then, cells were resuspended in 500 μl binding buffer and stained with Annexin V-FITC and PI according to the protocol. The cells were incubated in the dark at room temperature for 15 min. Finally, the percentage of apoptotic cells and necrotic cells were assessed by flow cytometer analysis (FACS Aria III Cell Sorter, BD, United States).

TUNEL staining was carried out to identify the rate of apoptotic cells. For the assay, cells inoculated on confocal dishes were fixed in 4% paraformaldehyde in PBS and permeabilized with 0.2% Triton X-100 in citrate buffer. Samples were incubated with TUNEL reaction mixture at 37 °C for 1 h, counterstained with Hoechst 33342, and observed with CLSM. The percentage of apoptotic cells was estimated by counting a total of 300 cells from random fields.

### 2.10 Caspase activity assay

CAL-27 cells were pre-treated with or without Z-VAD-FMK for 1 h and then co-cultured with NRC-03 for 4 h. Caspase-3 and caspase-8 activity were evaluated according to the manufacturer's instructions. Briefly, the cell lysate was added to the 96-well plates and incubated with 2 mM of the Ac-DEVD-pNA (caspase-3 substrate) or Ac-IETD-pNA (caspase-8 substrate) at 37 °C overnight. The absorbance was read at 405 nm in Multiskan™ FC Microplate Photometer (Thermo Scientific). Protein levels in the cell lysate were measured using a Bradford protein assay kit. The results were expressed as active units of caspase/μg protein. Relative caspase activity was expressed as a fold increase over the control.

### 2.11 Oxygen consumption rate evaluation

The oxygen consumption rate (OCR) was evaluated using an oxygen consumption rate assay kit. Briefly, CAL-27 cells were cultured in a 96-well plate (8 × 10^4^ cells/well) with a clear bottom and black sides for 24 h. Next, 100 μl of medium mixed with different concentrations of NRC-03 and 5 μl of oxygen fluorescent probe was added to each well. Thereafter, blocking buffer (2 drops/well) was immediately added to each well to prevent external oxygen generation. After that, the plate was read with a fluorescent microplate reader (Model Infinite 200 Pro, Tecan) at 37 °C (1 read per 3 min, Ex 455/Em 603). Since the fluorescence of this oxygen probe can be quenched by O_2_, the value of the fluorescence signal was inversely proportional to the amount of O_2_ in each well. OCR was calculated based on the changes of fluorescence signal over 2 h as follows: OCR (%) = (final fluorescence in NRC-03-treated cells−initial fluorescence in NRC-03-treated cells)/(final fluorescence in control cells−initial fluorescence in control cells) × 100%.

### 2.12 Measurement of cellular oxidative stress

The determination of intracellular oxidant stress was based on the oxidation of DCFH-DA. Briefly, CAL-27 cells were seeded in 60 mm dishes and treated with NRC-03 in the absence or presence of other test compounds for the indicated time, and then were incubated with redox-sensitive dye DCFH-DA at 37 °C for 30 min and analyzed by a flow cytometer.

For determining mitochondrial ROS production, CAL-27 cells were cultured in confocal dishes and treated with or without other test compounds for 1 h prior to NRC-03 for 4 h. The cells were stained with 100 nM Mitotracker green for 30 min, and then with 2 μM MitoSOX Red (excitation: 510 nm, emission: 580 nm) for 15 min at 37 °C and visualized with CLSM. The ratio of fluorescence intensities was determined by ImageJ software.

For determining hydrogen peroxide (H_2_O_2_) production, CAL-27 cells were cultured in a 96-well plate (8 × 10^4^ cells/well) with a clear bottom and black sides for 24 h. The cells were treated with or without other test compounds for 1 h prior to NRC-03 treatment for 4 h. The quantification of H_2_O_2_ production was assessed by AbIR Peroxidase Indicator using a Hydrogen peroxide assay kit – (Fluorometric-Near Infrared) complemented with a fluorescence plate reader (Model Infinite 200 Pro, Tecan) at room temperature (Ex 640/Em 680).

### 2.13 Mitochondrial membrane potential assay

CAL-27 cells were plated in 60 mm dishes and grown overnight. Cells were then treated for 2 h with NRC-03 at the indicated concentrations or vehicle control in the absence or presence of other test compounds. Cells were then stained with 1 μM JC-1 dye for 20 min at 37 °C, washed, and assessed via flow cytometer analysis.

### 2.14 Measurement of cellular ATP level

For the measurement of ATP level, whole-cell extracts were lysed in the lysis buffer provided in the ATP assay kit. After centrifugation at 12,000×*g* for 5 min at 4 °C, the supernatants were transferred to a new 1.5-ml tube for ATP analysis. The luminescence from a 100 μl sample was assayed in a luminometer (Model Infinite 200 Pro, Tecan) together with 100 μl of ATP detection buffer. A standard curve of ATP concentrations (1 nM–1 μM) was prepared from a known amount.

### 2.15 Western blot analysis

After the indicated treatments, cells were collected and lysed in cell lysis buffer containing a 1% protease inhibitor. Total protein concentrations were measured using a BCA protein assay kit. Proteins were separated by electrophoresis and transferred to a polyvinylidene difluoride (PVDF) membrane. The membranes were blocked with blocking buffer and then incubated overnight at 4 °C with the primary antibody, followed by incubation with the secondary antibody at room temperature. Protein bands were visualized with an enhanced chemiluminescence substrate, detected using the Molecular Imager Gel Doc XR + imaging system (Bio-Rad, United States), and quantified with Quantity One Software.

### 2.16 RNA sequencing

The total RNA of CAL-27 cells treated for 4 h with or without NRC-03 was isolated using an RNeasy mini kit (Qiagen, Germany). Paired-end libraries were synthesized by using the TruSeq RNA Sample Preparation Kit (Illumina, USA) following TruSeq RNA Sample Preparation Guide. Purified libraries were quantified by Qubit 2.0 Fluorometer (Life Technologies, USA) and validated by Agilent 2100 bioanalyzer (Agilent Technologies, USA) to confirm the insert size and calculate the mole concentration. A cluster was generated by cBot with the library diluted to 10 pM and then sequenced on the Illumina NovaSeq 6000 (Illumina, USA) by Promegen Biotechnology Co., Ltd, Guangzhou, China. The reads were aligned with Hisat2 (v 2.1.0) to GRCm38 with default parameters [33]. The output SAM (sequencing alignment/map) files were converted to BAM (binary alignment/map) files and sorted using SAMtools (version 1.3.1) [34]. Gene abundance was expressed as fragments per kilobase of exon per million reads mapped (FPKM). StringTie software was used to count the fragment within each gene, and the TMM algorithm was used for normalization [35]. Differential expression analysis for mRNA was performed using R package edgeR. Differentially expressed RNAs with |log2(FC)| value > 1 and q value < 0.05, considered as significantly modulated, were retained for further analysis.

### 2.17 RT-qPCR analysis

To further investigate the effect of NRC-03 on the apoptosis and mitochondrial oxidative stress-related gene expression of OSCC cells, CAL-27 cells treated for 4 h with or without NRC-03 were collected and total RNAs were extracted for RT-qPCR. Following the manufacturer's instructions, complementary DNA was synthesized from 500 ng of total RNA using a Takara PrimeScript™ RT Master Mix in T100 Thermal Cycler (Bio-Rad, United States). The cDNA was assayed using TaKaRa TB Green™ Premix Ex Taq™ in the CFX96 Real-Time system (Bio-Rad, United States). GADPH was used as a reference gene. The primers used for RT-qPCR are listed in Supplementary Table S1.

### 2.18 Animal xenograft model

Female BALB/c nu/nu mice (5-weeks-old) were purchased from the Center for Experimental Animals, Southern Medical University, Guangzhou, China. All animal studies were conducted in accordance with the guidelines of the National Regulation of China for Care and Use of Laboratory Animals (South China Normal University, Guangzhou, China). Southern Medical University Experimental Animal Ethics Committee approved all animal care and study protocols (L2018153). Mice were subcutaneously injected in the right flank with 4 × 10^6^ CAL-27 cells in 0.1 ml sterile PBS. At 2–4 weeks post-inoculation, tumors grew to an average volume of 100 mm^3^ and the CAL-27 tumor-bearing mice were randomly distributed into two groups (*n* = 6 per group) and intratumor injected with 50 μl PBS vehicle or 125 μg NRC-03 in 50 μl PBS. Treatments were repeated every other day for 15 days. Mice were monitored daily for tumor growth (using digital calipers), cachexia, and weight loss. Tumor volumes were calculated using the elliptical formula: 1/2 (length × width^2^). Some tumors were frozen in liquid nitrogen for western blot and the other part tumors and the organs including the heart, liver, spleen, lungs, and kidneys were formalin-fixed and processed for histological analysis.

Hematoxylin and eosin (H&E) staining, Ki-67 staining, and TUNEL staining were performed to detect proliferating cells and apoptotic cells respectively.

### 2.19 Statistical analysis

All data were representative results from at least three independent experiments and mean ± SD. Statistical analysis was performed using one-way analysis of variance (ANOVA) and unpaired Student's *t*-test by Graphpad Prism 6.0. *p* < 0.05 was considered statistically significant.

## 3 Results

### 3.1 NRC-03 induced apoptosis in oral squamous cell carcinoma cells

**Fig. 1:**
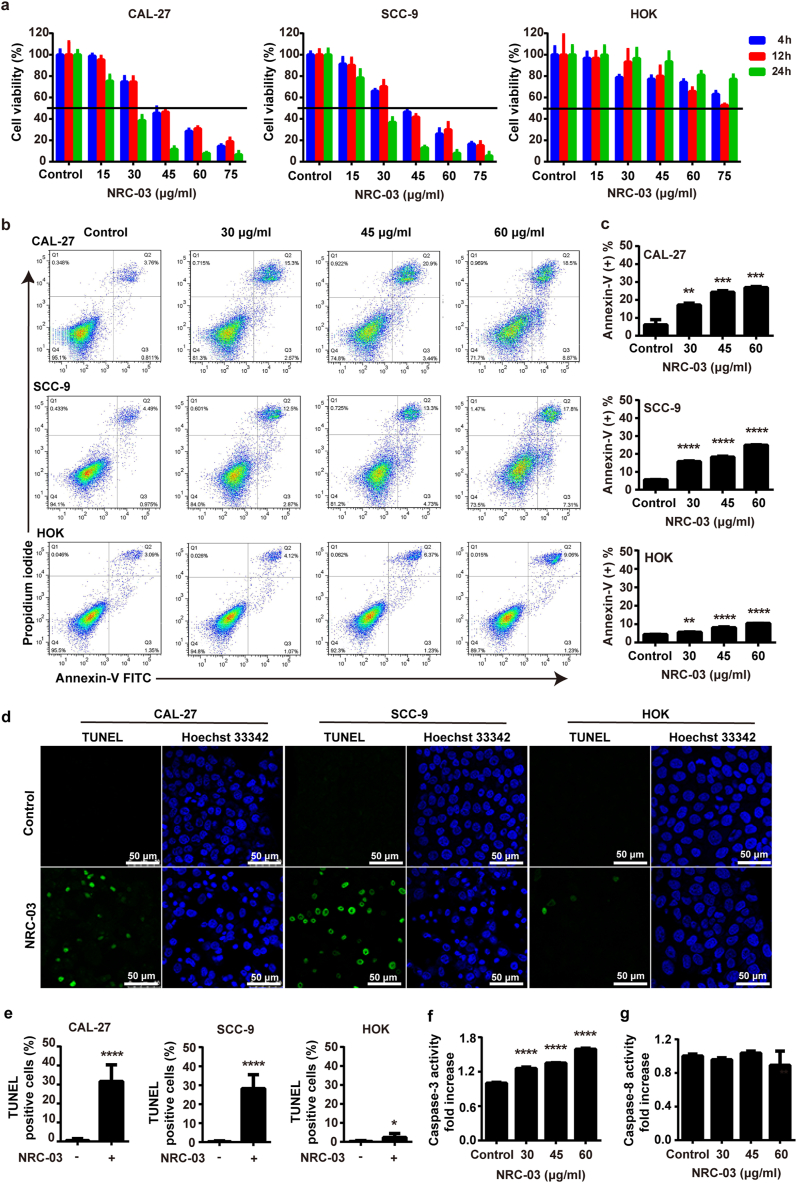
**NRC-03 induced apoptosis in oral squamous cell carcinoma cells**. **(a)** Cell viability of NRC-03-treated OSCC cells CAL-27 and SCC-9, and HOK analyzed by CCK-8 assay (*n* = 4). NRC-03 treatment for 4 h induced apoptosis in OSCC cells. **(b)** Detection of phosphatidylserine exposure by Annexin-V FITC/PI staining and analysis by flow cytometry. Cells in Q1, Q2, Q3, and Q4 respectively represent cell debris (Annexin-V^-^/PI^+^), cells in late apoptosis (Annexin-V^+^/PI^+^), cells in early apoptosis (Annexin-V^+^/PI^−^), and healthy cells (Annexin-V^-^/PI^−^). **(c)** The quantification of apoptotic cells (Annexin-V^+^) (*n* = 3). **(d)** Representative immunofluorescence images of TUNEL staining of cells treated with 45 μg/ml NRC-03. **(e)** Quantification of TUNEL positive cells (*n* = 6). **(f)** Caspase-3 activity in NRC-03-treated CAL27 cells (*n* = 3). **(g)** Caspase-8 activity in NRC-03-treated CAL-27 cells (*n* = 3). Data are presented as mean ± SD. Significant difference compared with the control group, *p < 0.05, **p < 0.01, ***p < 0.001, and ****p < 0.0001.

CCK-8 assay showed that NRC-03 in the concentration range of 15–75 μg/ml inhibited the viability of CAL-27 and SCC-9 cells in a time- and dose-dependent manner (Fig. 1a). Cytotoxicity of all the tested concentrations of NRC-03 toward HOK cell was minimum compared with CAL-27 or SCC-9 cells (Fig. 1a). Annexin/propidium iodide staining assay is an indicator of alterations in cell membrane permeability and apoptosis. Annexin/propidium iodide staining revealed a dose-dependent increase in apoptosis in NRC-03-treated CAL-27 and SCC-9 cells. NRC-03 (30–60 μg/ml) significantly enhanced the rate of apoptosis in CAL-27 and SCC-9 cells by about 2.8–4.4-fold. In contrast, the enhancement magnitude of HOK apoptosis was much lower (about 1.2–2.3-fold) (Fig. 1b and 1c). The pro-apoptotic effects of NRC-03 were further verified by TUNEL staining as an indicator of DNA damage (Fig. 1d). The percentage of TUNEL positive cells significantly increased in OSCC groups, i.e., 31.61% ± 7.98% in CAL-27 and 28.25% ± 6.64% in SCC-9 cells, respectively (Fig. 1e), while TUNEL staining positivity rate was only 2.37% ± 1.90% in HOK cells after treatment with 45 μg/ml NRC-03. In contrast, it is important to note that NRC-03 did not substantially affect the viability or induce apoptosis in the normal HOK cells (Fig. 1a, 1d, and 1e). Caspase-3 upregulates during apoptosis-mediated cell death. Caspase-8 is a crucial initiator in the death receptor-mediated apoptotic pathway. Next, we detected the caspase-3 and caspase-8 activity in NRC-03-treated CAL-27 cells. NRC-03 treatment didn't activate the caspase-8 activity but caused a remarkable increase in caspase-3 activity of OSCC cells (Fig. 1f and 1g). The median lethal dose of NRC-03 treatment was 45 μg/ml on 4 h treatment. NRC-03 at a concentration of 45 μg/ml significantly inhibited the cell viability of CAL-27 and SCC-9 cells on 24 h treatment, but the effect on normal HOK viability was not that prominent (Fig. 1a). Based on these findings, 45 μg/ml of NRC-03 was chosen as the optimal dose to treat the cells in subsequent experiments.

**Fig. 2:**
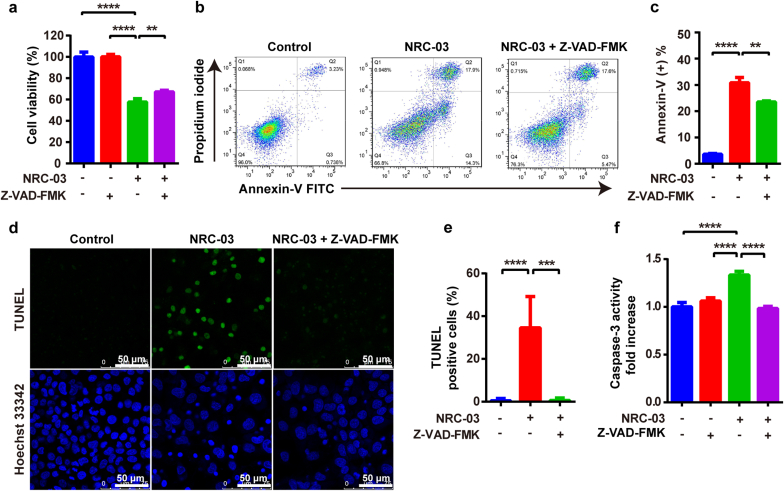
**Caspase inhibition attenuated NRC-03-induced apoptosis in CAL-27 cells.** CAL-27 cells were cultured in the presence of 45 μg/ml NRC-03 for 4 h with or without caspase inhibitor Z-VAD-FMK (50 μM). **(a)** Cell viability determined by CCK-8 (*n* = 4). **(b)** Flow cytometry analysis of annexin-V positive cells. **(c)** Quantification of annexin-V positive cells (*n* = 3). **(d, e)** TUNEL immunofluorescence staining and quantification of TUNEL positive cells (*n* = 5). **(f)** Caspase-3 activity (*n* = 3). Data are presented as mean ± SD. Significant difference between the groups, **p < 0.01, ***p < 0.001, and ****p < 0.0001.

To further confirm the NRC-03-mediated apoptotic pathway in OSCC cells, we pretreated CAL-27 cells with Z-VAD-FMK, an irreversible pan-caspase inhibitor, along with NRC-03. Z-VAD-FMK treatment counteracted the NRC-03-induced cytotoxicity (Fig. 2a) and apoptosis (Fig. 2b–e). Furthermore, Z-VAD-FMK nullified the promoting effects of NRC-03 on the caspase-3 activity in CAL-27 cells (Fig. 2f). Collectively, these data verified that NRC-03 induced OSCC cell death mainly via the intrinsic/mitochondria-mediated apoptosis pathway.

### 3.2 NRC-03 inhibits tumor growth in a xenograft model

**Fig. 3:**
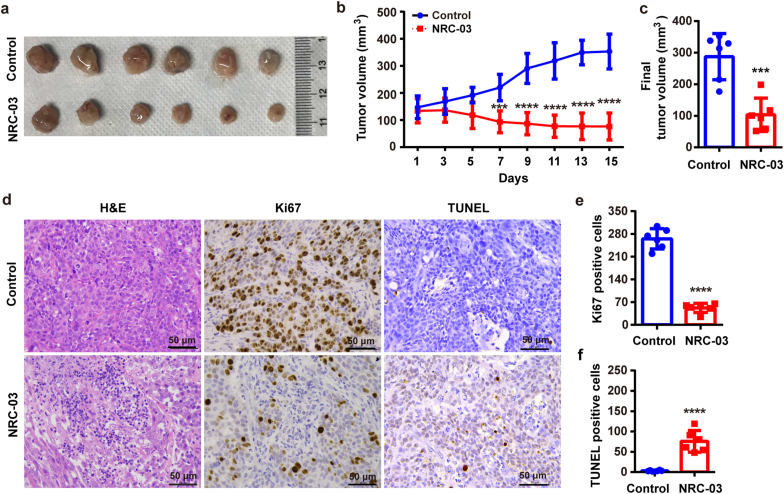
**NRC-03 inhibited tumor growth in the ectopic tumor model of OSCC.** CAL-27-derived xenografts were treated with 125 μg NRC-03 every other day for 15 days. **(a)** Gross images of representative tumor tissues on day 15. **(b)** Tumor volume at different time points (*n* = 6). **(c)** Final tumor volume on day 15 (*n* = 6). **(d)** Representative microscopic images of tumor tissue sections showing tumor morphology (H&E staining), proliferation (Ki-67 immunohistochemistry), and apoptosis (TUNEL staining). Quantification of Ki-67-positive cells **(e)** (*n =* 6), **and** apoptotic cells **(f)** (*n =* 6). Data are presented as mean ± SD. Significant difference compared with the respective control group, ***p < 0.001, and ****p < 0.0001.

NRC-03 at a dose of 125 μg/animal inhibited the growth of CAL-27-derived tumors in a subcutaneous ectopic tumor model in nude mice (Fig. 3). Importantly, NRC-03 did not cause observable damage to vital organs (Fig. S1a) or bodyweight (Fig. S1b). By day 9, compared with the vehicle control, NRC-03 treatment induced a significant decrease in tumor growth (*p* < 0.01), which persisted throughout the 15-day study period (Fig. 3b). The final average tumor volumes in the control and 125 μg NRC-03 treated groups were 287.18 ± 66.73 mm^3^ and 103.17 ± 48.16 mm^3^, respectively (Fig. 3c). Moreover, tumor tissues from the NRC-03-treated group showed a reduced cellular density and proliferation rate (Fig. 3d and e) and increased apoptosis rate (Fig. 3d and f) compared with the control group. These results *in vivo* are consistent with the results from the *in-vitro* studies, which highlighted that NRC-03 could inhibit OSCC growth via inducing apoptosis in cancer cells.

### 3.3 NRC-03 damaged the cell membrane and specifically entered the cytoplasm and nucleus of CAL-27 cells

**Fig. 4:**
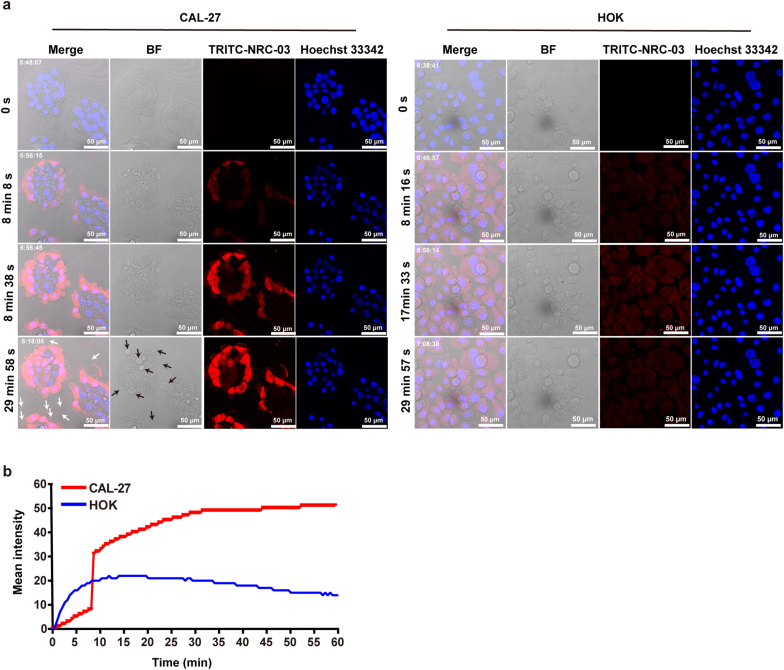
**NRC-03 damaged the cell membrane of CAL-27 cells and entered the cytoplasm and nucleus.** CLSM images of cells were treated with 45 μg/ml NRC-03 for 1 h and photographed by CLSM every 30 s. **(a)** Peptide and nucleus were visualized by using TRITC-NRC-03 (Red) and Hoechst 33342 (Blue), respectively. The white arrow indicates extruded peptide-bound substance from CAL-27 cells. The black arrow indicates membrane blebs on the cell surface of CAL-27. **(b)** Quantification of the mean fluorescence intensity of TRITC-NRC-03 at different time points. (For interpretation of the references to color in this figure legend, the reader is referred to the Web version of this article.)

To investigate whether NRC-03 entered the cytoplasm of OSCC cells, CAL-27 or HOK cells were treated with TRITC labeled NRC-03 for 1 h and monitored continuously by CLSM. CLSM revealed that TRITC labeled NRC-03 rapidly entered the cytoplasm of CAL-27 cells (Fig. 4a). The dynamic process of NRC-03 entering the cell can be seen in the supplementary information (Supplementary Movie 1 and 2). A remarkably increased accumulation of NRC-03 was observed at 8–9 min in CAL-27 cells, which was 1.56-fold higher than that in HOK cells at the same time. With the prolongation of treatment time, the accumulation of NRC-03 gradually increased and stabilized at 30 min in CAL-27. However, the cellular accumulation of NRC-03 reached its maximum at 17–18 min in HOK and then gradually decreased. Moreover, the amount of NRC-03 in CAL-27 cells was 3.48-fold higher than in HOK cells at 60 min (Fig. 4b). Importantly, NRC-03 localization in the nucleus of CAL-27 cells was higher than that in HOK cells. The cell surface of CAL-27 showed a higher accumulation of NRC-03 forming many membrane blebs as indicated with black arrows (Fig. 4a). Moreover, some CAL-27 cells extruded a peptide-bound substance as indicated with white arrows (Fig. 4a), suggesting that the peptides cause significant membrane damage to CAL-27 cells. Taken together, these results demonstrated that NRC-03 selectively targets OSCC cells, causes membrane blebbing, and localizes in the nucleus.

Supplementary video related to this article can be found at https://doi.org/10.1016/j.redox.2022.102355.

The following are the supplementary data related to this article:

Movie 1 Movie 1

Movie 2 Movie 2

### 3.4 NRC-03 bound to mitochondria and changed mitochondrial morphology of CAL-27 cells

**Fig. 5:**
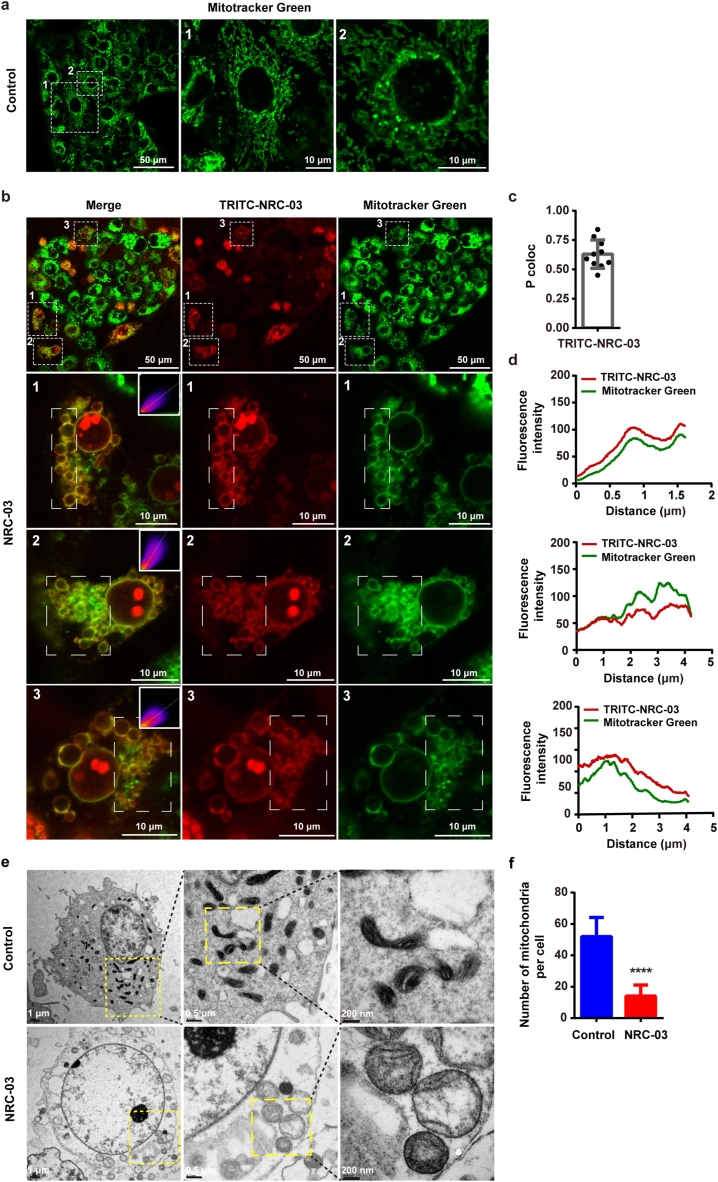
**NRC-03 bound to mitochondria and changed the mitochondrial morphology of CAL-27 cells.** CAL-27 cells were treated with or without 45 μg/ml NRC-03 for 30 min. The mitochondria and peptides were visualized with CLSM by using Mitotracker Green (Green) and TRITC-NRC-03 (Red) respectively. **(a)** The mitochondria of CAL-27 cells without NRC-03 treatment. **(b)** Co-localization of NRC-03 and mitochondria in NRC-03-treated CAl-27 cells. An embedded scatter plot (upper right corner on the merged image) estimates the amount of each detected antigens based on colocalization in mitochondria (Green, y-axis) and peptide (Red, x-axis) in the white dotted frame. Colocalized pixels of yellow color were located along the diagonal of the scatter plot. **(c)** The Pearson's correlation coefficient of mitochondria and peptides from 10 cells was calculated by the Image J colocalization analysis plugin. Error bars indicate SD (*n* = 10). **(d)** Fluorescence signals analysis based on the white dotted frame in figure **(b)**. CAL-27 cells and the mitochondria were visualized with TEM. **(e)** Morphology of mitochondria in CAL-27 cells. **(f)** The number of mitochondria per cell was counted (*n* = 6). Data are presented as mean ± SD. The significant difference compared with the respective control group, ****p < 0.0001. (For interpretation of the references to color in this figure legend, the reader is referred to the Web version of this article.)

The mitochondria and NRC-03 were visualized with CLSM using MitoTracker green and TRITC-NRC-03 red respectively. The localization of NRC-03 in mitochondria was illustrated by a computed fluorescence intensity profile based on the white dotted frame in a fluorescence image of Fig. 5b. According to the profile analysis, we observed a prominent overlap between the MitoTracker Green signals and TRITC-NRC-03 red signal (Fig. 5d), suggesting that NRC-03 could localize in the mitochondria of CAL-27 cells. To confirm the data obtained in the fluorescence intensity profile analysis, we calculated the degree of co-localization (Pearson's correlation coefficient) of mitochondria and NRC-03. The result showed that NRC-03 co-localized with mitochondria with a P coloc value of 0.63 ± 0.12 (Fig. 5c). In addition, NRC-03-treated cells showed a different morphological pattern of mitochondria compared with the control group. Mitochondrial morphology was changed from a long rod shape (Fig. 5a) to a spherical shape (Fig. 5b) by the effect of NRC-03. The large, swollen mitochondria in the NRC-03-treated group were observed by using TEM (Fig. 5e). Moreover, compared to the control group, the number of mitochondria per cell was significantly reduced in the NRC-03-treated group (Fig. 5f). To sum up, these data indicate that NRC-03 targets mitochondria and alters the mitochondrial morphology of OSCC cells.

Similarly, the lysosomes and NRC-03 were visualized using LysoTracker Green and TRITC-NRC-03 red respectively. The localization of NRC-03 in lysosomes was illustrated by a computed fluorescence intensity profile based on the white dotted frame in a fluorescence image in Fig. S2a. The waveforms and trends of the LysoTracker Green signal and TRITC-NRC-03 red signal were inconsistent (Fig. S2b), suggesting that NRC-03 could not co-localize with lysosomes in CAL-27 cells.

### 3.5 NRC-03 treatment induced mitochondrial oxidative stress and mitochondrial dysfunction in CAL-27 cells

**Fig. 6:**
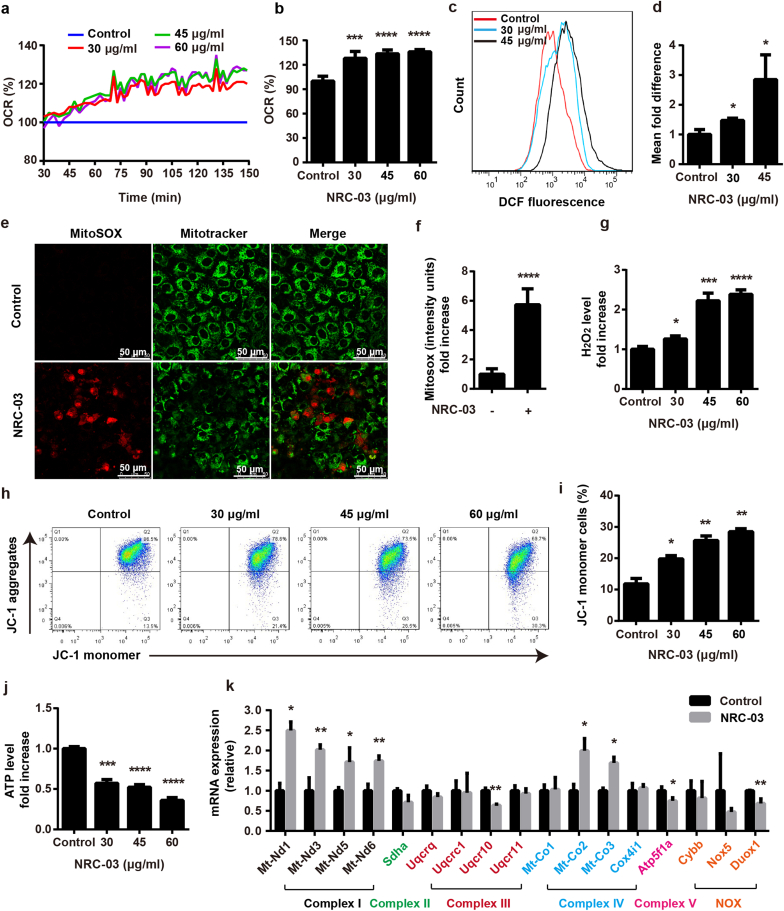
**NRC-03 induced mitochondrial dysfunction in CAL-27 cells. (a)** The rate of oxygen consumption at different time points. Data are presented as mean (*n* = 5). **(b)** The rate of oxygen consumption at 131 min (*n* = 5). **(c, d)** Flow cytometry analysis of intracellular ROS generation in CAL-27 cells using redox-sensitive dye DCFH-DA (*n* = 3). **(e, f)** MitoSOX staining and quantification (*n* = 5). **(g)** H_2_O_2_ production in the indicated groups (*n* = 3). **(h, i)** The mitochondrial membrane potential determined by flow cytometry of JC-1-stained cells (*n* = 3). **(j)** ATP production in the indicated groups (*n* = 3). **(k)** Relative mRNA expression of mitochondrial respiratory chain complexes and NOX-related genes analyzed by RT-qPCR (*n* = 3). For figure **c**–**k**, CAL-27 cells were treated by NRC-03 for 4 h. Data are presented as mean ± SD. Significant difference compared with the respective control group, *p < 0.05, **p < 0.01, ***p < 0.001, and ****p < 0.0001.

To determine the effect of NRC-03 on oxygen metabolism, OCR in CAL-27 cells was evaluated. The value of the fluorescence signal of the oxygen probe was decreased in NRC-03-treated cells (Fig. S3). This result showed that NRC-03 caused rapid oxygen consumption in CAL-27 cells. Irrespective of concentration, NRC-03 significantly induced a rapid increase of OCR in CAL-27 cells after a 30 min treatment and reached a plateau level at 90 min (Fig. 6a). The oxygen consumption reached the highest level (around 28% more than Control) at 131 min in NRC-03-treated CAL-27 cells (Fig. 6b). Mitochondria are the primary source of ROS and the principal sites of ROS-induced damage. NRC-03 markedly increased the production of total intracellular ROS as indicated by the results of flow cytometry analysis using DCFH-DA (Fig. 6c and d). Similarly, mitochondrial ROS (mtROS) was also upregulated in CAL-27 cells by the effect of NRC-03 treatment as indicated by the results of MitoSOX staining (Fig. 6e and f). Moreover, NRC-03 significantly elevated the level of H_2_O_2_ in CAL-27 cells (Fig. 6g). NRC-03 treatment reduced the mitochondrial membrane potential as indicated by flow cytometry analysis using JC-1 staining (Fig. 6h and i). The intracellular ATP level was aslo significantly reduced in NRC-03-treated CAL-27 cells (Fig. 6j). Superoxide (O_2_^•^^−^) and H_2_O_2_ are the most well studied ROS in cancer [36]. O_2_^•^−is formed from molecular O_2_ by accepting a single electron from the mitochondria at complex I and III of the respiratory chain or from NADPH oxidases (NOX) [37]. O_2_^•^−is further converted into H_2_O_2_ by superoxide dismutase enzymes [38]. Since endogenous ROS is mainly produced by complex I, III, and NOX. Consequently, our findings suggested that the mitochondrial respiratory chain might be damaged by NRC-03. With this inspiration, we further adopted RT-qPCR to assess the mRNA expression of the genes encoding the key subunits of mitochondrial respiratory chain complexes and NOX in the CAL-27 cells in the absence or presence of 45 μg/mL NRC-03. As shown in Fig. 6k, the mRNA expression levels of genes encoding complex I subunits *Mt-Nd1*, *Mt-Nd3, Mt-Nd5,* and *Mt-Nd6* were significantly increased (1.7–2.5-fold) in NRC-03-treated CAL-27 cells in comparison with the control cells. In contrast, the mRNA expression levels of *Uqcr10* (complex III subunit X) and *Duox1* (a member of the NOX family) were significantly decreased by 36.2%, and 31.3% respectively. These results suggested that the excessive ROS was mainly attributed to complex I. Complex IV catalyzes the reduction of oxygen to water. Complex IV genes *Mt-Co2,* and *Mt-Co3* were upregulated which might be a defense mechanism against excessive ROS. Consistent with the significant decrease in ATP production, the mRNA expression level of *Atp5f1a*, a key subunit of complex V that catalyzes ATP synthesis was downregulated by 24.9%. These data suggested that the dysfunctions of complex III and upregulated complex I might be responsible for the NRC-03-induced decrease in ATP production and increase of mtROS. All these data suggested that NRC-03 triggers mitochondrial oxidative stress and mitochondrial dysfunction through elevating oxygen consumption and upregulation of mitochondrial respiratory chain complex I.

### 3.6 Inhibition of cellular and mitochondrial ROS attenuated NRC-03-induced apoptosis and mitochondrial dysfunction in CAL-27 cells

**Fig. 7:**
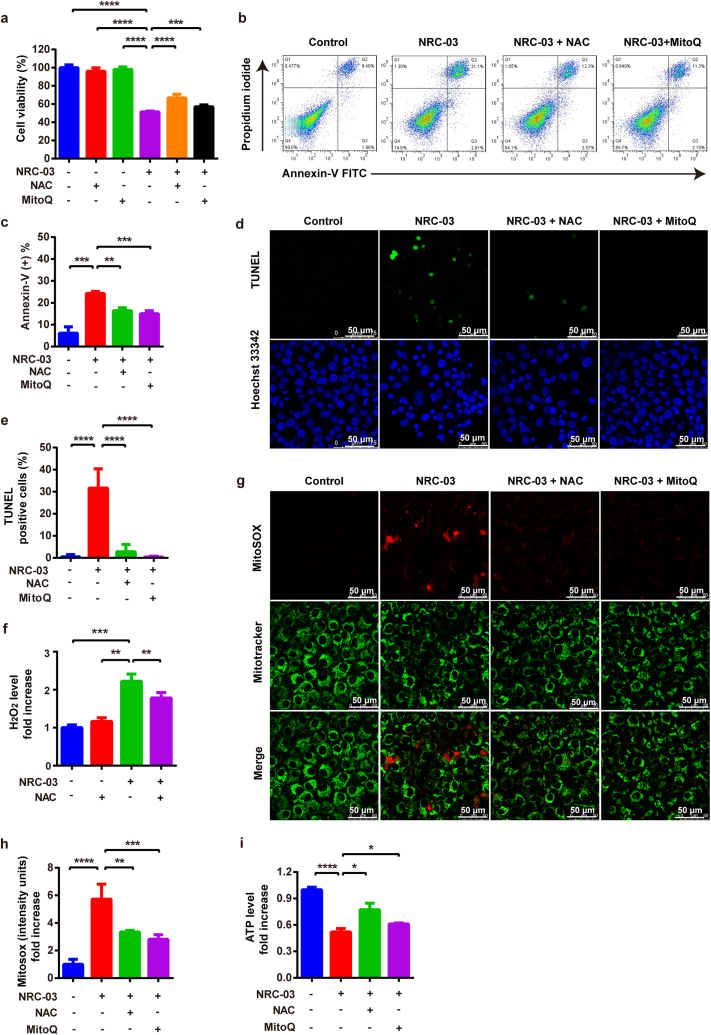
**Inhibition of cellular and mitochondrial ROS attenuated NRC-03-induced apoptosis and mitochondrial dysfunction in CAL-27 cells.** CAL27 cells were treated with or without antioxidant NAC (5 mM) or MitoQ (1 μM) for 4 h in presence of NRC-03 (45 μg/ml). **(a)** Cell viability determined by CCK-8 (*n* = 5). **(b, c)** Flow cytometry analysis of apoptosis (*n* = 3). (**d, e)** TUNEL immunofluorescence staining and quantification of TUNEL positive cells (*n* = 5). **(f)** H_2_O_2_ production in the indicated groups (*n* = 3). **(g, h)** MitoSOX staining and quantification (*n* = 5). **(i)** ATP production in the indicated groups (*n* = 3). Data are presented as mean ± SD. Significant difference between the respective groups, *p < 0.05, **p < 0.01, ***p < 0.001, and ****p < 0.0001.

To further determine the role of mitochondrial oxidative stress in NRC-03-induced cell apoptosis and mitochondrial dysfunction, we pretreated CAL-27 cells with a non-specific antioxidant (NAC), or a mitochondria-specific antioxidant (MitoQ), along with NRC-03. Compared with those only treated with NRC-03, NAC and MitoQ partially restored the viability of the CAL-27 cells (Fig. 7a) and significantly alleviated cell apoptosis induced by NRC-03 (Fig. 7b–e). NAC decreased the H_2_O_2_ production caused by NRC-03 (Fig. 7f). In addition, both NAC and MitoQ antagonized the effects of NRC-03 by significantly suppressing NRC-03-induced mtROS (Fig. 7g and h) and elevating the NRC-03-inhibited ATP level in CAL-27 cells (Fig. 7i). NAC and MitoQ demonstrated efficient anti-oxidative and mitochondria-protective effects against NRC-03. In summary, these results further supported that mitochondrial oxidative stress was involved in NRC-03-induced OSCC cells apoptosis and mitochondrial dysfunction.

### 3.7 RNA-seq verified the involvement of mitochondrial dysfunction, oxidative stress, and apoptosis in NRC-03-induced CAL-27 cell death

**Fig. 8:**
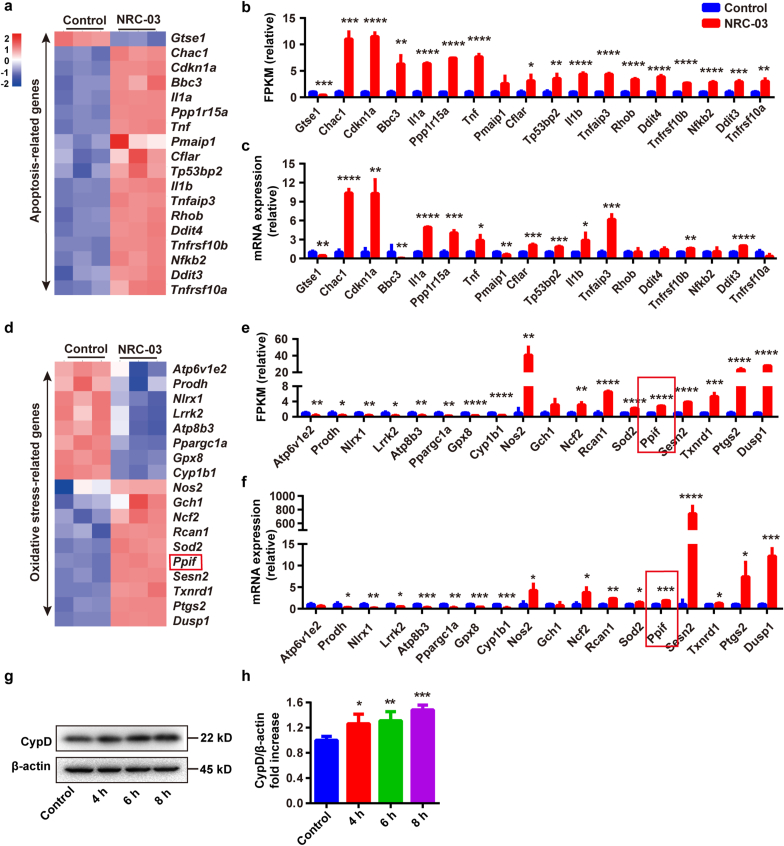
**NRC-03-treatment altered the expression pattern of apoptosis and oxidative stress-related genes in CAL-27 cells. (a)** Heatmap of differentially expressed apoptosis-related genes analyzed by RNA-seq. **(b)** Quantification of differentially expressed apoptosis-related genes analyzed based on FPKM. (**c)** Relative mRNA expression of apoptosis-related genes analyzed by RT-qPCR (*n* = 3). **(d)** Heatmap of differentially expressed oxidative stress-related genes analyzed by RNA-seq. **(e)** Quantification of differentially expressed oxidative stress-related genes analyzed based on FPKM. (**f)** Relative mRNA expression of oxidative stress-related genes analyzed by RT-qPCR (*n* = 3). **(g, h)** Western blot analysis of CypD protein in CAL-27 cells with or without NRC-03 (45 μg/ml) treatment (*n* = 4). Data are presented as mean ± SD. Significant difference compared with the respective control group, *p < 0.05, **p < 0.01, ***p < 0.001, and ****p < 0.0001.

Whole transcriptome RNA sequencing was conducted to confirm the aforementioned results. A pairwise comparison was implemented to determine the expression profile among Control and NRC-03 groups. NRC-03-treated CAL-27 cells showed 752 differentially upregulated and 1129 differentially downregulated genes compared with control CAL-27 cells (Fig. S4a). The genes involved in mitochondrial function (Figs. S4b and S4c) were further analyzed. Among these genes, *Cox6b2* was upregulated 8-fold in NRC-03-treated CAL-27 cells*. Cox6b2* is an encoding subunit of the mitochondrial respiratory complex IV. Cytochrome P450 superfamily genes *Cyp2u1*, *Cyp1a1*, and *Cyp27b1* were differentially expressed in NRC-03-treated CAL-27 cells. Cytochrome P450 enzymes regulated the metabolism of a large number of endogenous and exogenous compounds and ROS production [39,40]. Moreover, the members of the mitochondrial solute carrier family *Slc25a21*, *Slc25a25,* and *Slc25a27* were downregulated. The SLC25 family transport proteins are essential for many cellular processes related to the mitochondrial inner membrane energy conversion and maintenance of the cells [41]. Other genes related to ATP synthesis and decomposition i.e., *Atp1b1*, *Atp6v1b1*, *and Atpaf1* were differentially expressed in NRC-03-treated CAL-27 cells. These results demonstrated that NRC-03 altered mitochondrial functions in OSCC cells. The GO pathway enrichment analysis indicated that upregulated genes were significantly enriched in stress-related signaling pathways (Fig. S4d), such as response to oxidative stress, stress-activated protein kinase signaling cascade, ERK1/2 cascade, and cell growth. While the downregulated genes were mainly enriched in cell division-related signaling pathways (Fig. S4e), such as organelle fission, nuclear division, regulation of cell cycle phase transition, and negative regulation of organelle organization. Then KEGG pathway classification analysis of these genes suggested that the most enriched metabolic pathways were located in signal transduction (Fig. S4f). Notably, the signaling pathways involved in signal transduction were mainly enriched in the MAPK signaling pathway, TNF signaling pathway, and NF-κB signaling pathway (Fig. S4g). The signaling pathways involved in cell growth and death were mainly enriched in the cell cycle, apoptosis, and cellular senescence (Fig. S4g). Next, we further confirmed the expression pattern of apoptosis (Fig. 8a–c) and oxidative stress-related genes (Fig. 8d–f) in CAL-27 cells by RT-qPCR. The results further confirmed that apoptosis-related genes and oxidative stress-related genes were significantly regulated in NRC-03-treated cells. Notably, we unraveled the >10.0-fold upregulation of pro-apoptotic factors genes *Chac1* and *Cdkn1a*, and oxidative stress-related genes *Sesn2* and *Dusp1*. Taken together, the results from RNA-seq and RT-qPCR analysis indicated that NRC-03 induced mitochondrial dysfunction, oxidative stress, cell apoptosis, and arrested cell cycle through regulation of MAPK, TNF, and NF-κB signaling pathways.

### 3.8 NRC-03-induced CypD expression regulated apoptosis and mitochondrial dysfunction in CAL-27 cells

**Fig. 9:**
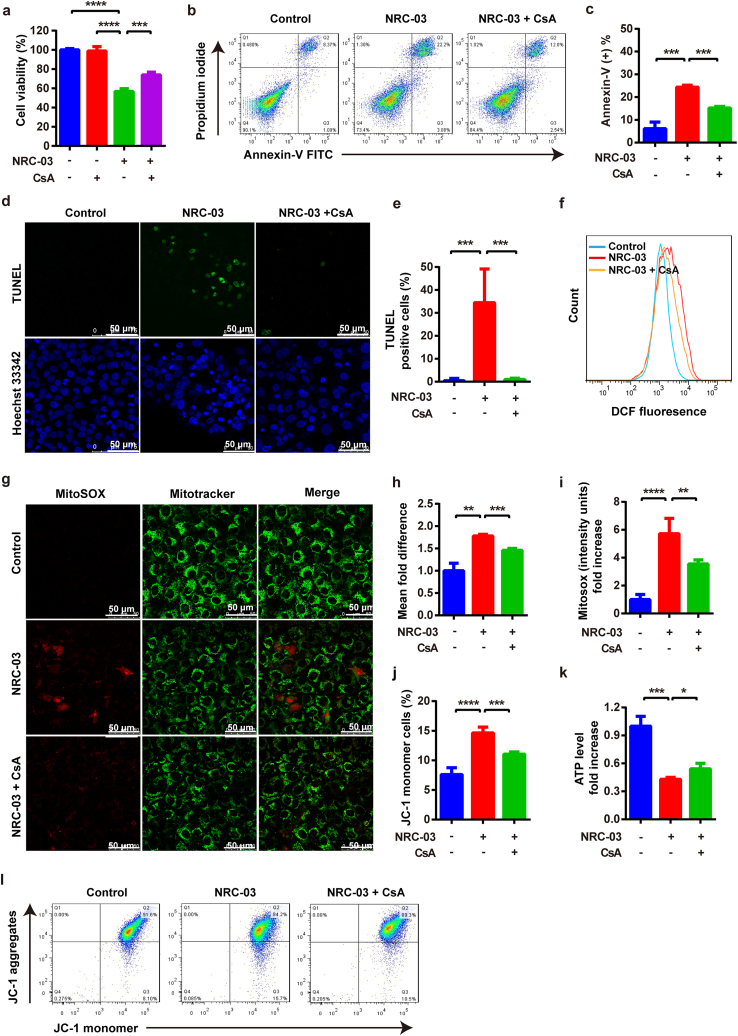
**Inhibition of CypD attenuated NRC-03-induced apoptosis and mitochondrial dysfunction in CAL-27 cells.** Cells were treated with or without CsA (2 μM) for 4 h in presence of NRC-03 (45 μg/ml). **(a)** Cell viability determined by CCK-8 (*n* = 4). **(b, c)** Flow cytometry analysis of apoptosis (*n* = 3). **(d, e)** TUNEL immunofluorescence staining and quantification of TUNEL positive cells (*n* = 5). **(f, h)** Flow cytometry analysis of intracellular ROS generation in CAL-27 cells using redox-sensitive dye DCFH-DA (*n* = 3). **(g, i)** MitoSOX staining and quantification (*n* = 5). **(k)** ATP production in the indicated groups (*n* = 3). **(j, l)** The mitochondrial membrane potential is determined by flow cytometry of JC-1-stained cells (*n* = 4). Data are presented as mean ± SD. Significant difference between the respective groups, *p < 0.05, **p < 0.01, ***p < 0.001, and ****p < 0.0001.

mPTP is a non-specific pore in the inner mitochondrial membrane. CypD, a crucial component of mPTP, is encoded by the *Ppif* gene and facilitates an opening of the mPTP and causes mitochondrial dysfunction, which is implicated in the regulation of cell death. Results from RNA-seq and RT-qPCR showed robust upregulation of *Ppif* in NRC-03-treated CAL-27 cells (Fig. 8d–f). Western blot analysis showed that NRC-03 significantly upregulated the expression of CypD in CAL-27 cells (Fig. 8g and h). We further analyzed the role of NRC-03-induced CypD overexpression in mitochondrial dysfunction and apoptosis. CsA, a pharmacological inhibitor of CypD inhibits mPTP formation by blocking the interaction of CypD with adenine nucleotide translocator (ANT). CsA treatment rescued the NRC-03-induced cytotoxicity (Fig. 9a) and apoptosis (Fig. 9b–e) in CAL-27 cells. Moreover, CsA decreased the NRC-03-induced intracellular ROS (Fig. 9f and h) and mtROS generation (Fig. 9g and i). Similarly, CsA restored NRC-03-disrupted mitochondrial membrane potential (Fig. 9j and l) and elevated the ATP level reduced by NRC-03 (Fig. 9k). All these observations implied that CypD-dependent mPTP could play a critical role in NRC-03-induced mitochondrial dysfunction and apoptosis of OSCC cells.

**Fig. 10:**
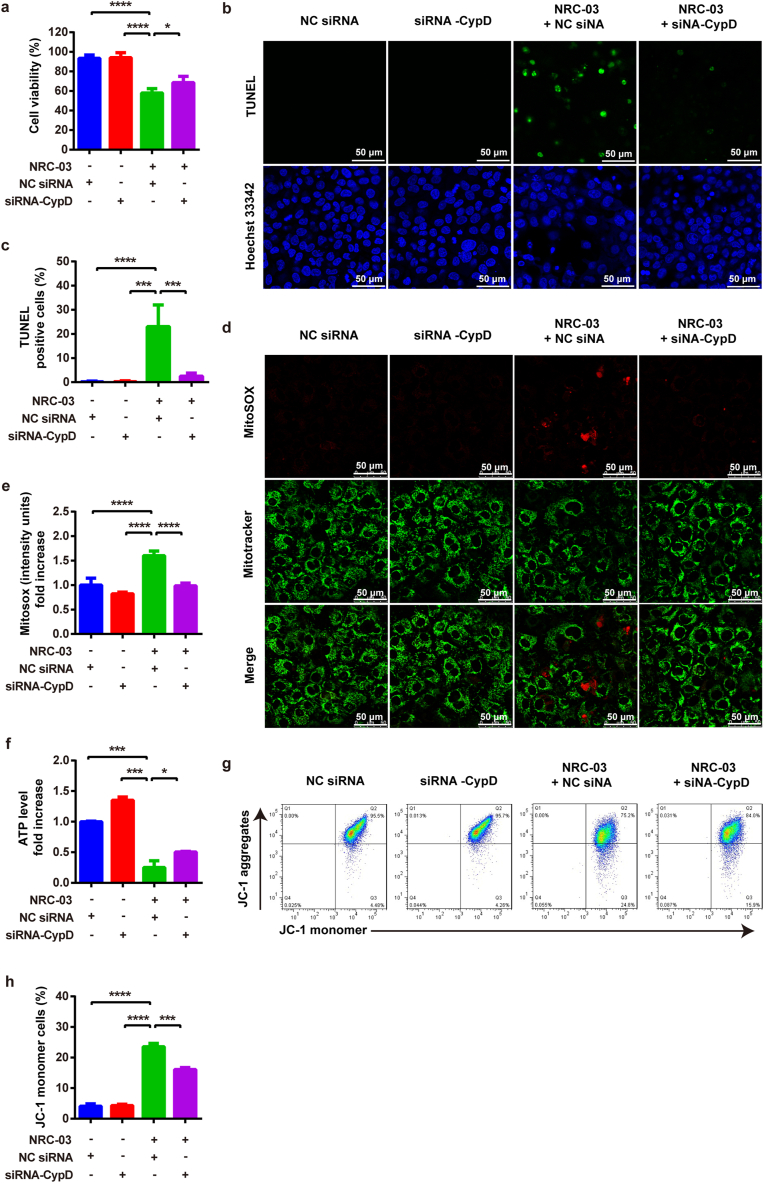
**Knockdown of CypD by siRNA attenuated NRC-03-induced apoptosis and mitochondrial dysfunction in CAL-27 cells.** Cells were treated with NC siRNA or siRNA-CypD for 4 h in presence of NRC-03 (45 μg/ml). **(a)** Cell viability determined by CCK-8 (*n* = 4). **(b, c)** TUNEL immunofluorescence staining and quantification of TUNEL positive cells (*n* = 6). **(d, e)** MitoSOX staining and quantification (*n* = 5). **(f)** ATP production in the indicated groups (*n* = 3). **(g, h)** The mitochondrial membrane potential determined by flowcytometry of JC-1-stained cells (*n* = 3). Data are presented as mean ± SD. Significant difference between the respective groups, *p < 0.05, ***p < 0.001, and ****p < 0.0001.

We knockdowned *Ppif* gene using siRNA-CypD to further confirm the role of CypD in NRC-03-mediated mitochondrial dysfunction and apoptosis of CAL-27 cells. siRNA-CypD significantly inhibited the expression of *Ppif* gene in CAL-27 cells (Fig. S5). siRNA-CypD rescued the NRC-03-induced cytotoxicity and apoptosis in CAL-27 cells (Fig. 10a–c). Moreover, siRNA-CypD reversed the NRC-03-induced mtROS (Fig. 10d and e), restored ATP level (Fig. 10f), and mitochondrial membrane potential (Fig. 10g and h). Our results confirmed that CypD-dependent mPTP plays a critical role in NRC-03-induced mitochondrial dysfunction and apoptosis of OSCC cells.

## 4 Discussion

An ideal chemotherapeutic drug to treat OSCC should be highly specific to cancer cells to minimize side effects and drug resistance. NRC-03 robustly inhibited the growth of OSCC cells both *in vitro* and *in vivo,* but with a mild effect on normal oral cells. NRC-03 more easily entered and accumulated in much larger quantities in the intracellular space of the OSCC cells than in HOK cells. We further showed that NRC-03 targeted mitochondria and triggered mitochondrial oxidative stress, mitochondrial dysfunction, and eventually cancer cell apoptosis. Importantly, the mRNA and protein expression of CypD, the key component of mPTP, was significantly upregulated in NRC-03-treated cancer cells, and the inhibition of CypD expression or function in cancer cells dramatically attenuated the NRC-03-induced mitochondrial oxidative stress, mitochondrial dysfunction, and apoptosis. Our findings indicated that NRC-03 substantially induces apoptosis in OSCC cells via the CypD-mPTP axis-mediated mitochondrial oxidative stress, suggesting a promising application potential of NRC-03 for OSCC treatment.

Unspecific targeting and death of rapidly dividing cells in the body are the major drawbacks of the current first-line chemotherapeutic drugs [6,13]. Such drawbacks cause a series of side effects and severely compromise the life quality of OSCC patients [42]. Furthermore, chemotherapy is also associated with the development of multidrug resistance [43]. Therefore, high selectivity and specificity to cancer cells are the most desired properties of novel anti-cancer chemotherapeutic drugs [44,45]. CAPs have attracted significant interest and have been proposed as a novel family of anticancer molecules over the past decade [46,47]. CAPs are small, amphipathic, and positively charged peptides present in all forms of life and comprise a major component of the innate immune system against various microbial pathogens [48]. CAPs can electrostatically bind to, and form pores, modify ion channels, or induce rupture on the negatively-charged cell membrane, causing microbial death [49]. This property confers CAPs a promising potential in treating cancers. In contrast to the neutral charge of normal cell membrane [50], the cancer cell membrane is negatively charged due to the exposure of anionic phospholipid phosphatidylserine and the presence of a greater abundance of anionic molecules such as heparan sulfate proteoglycans, sialic acid, and O-glycosylated mucins [16,[51], [52], [53]]. After binding with the cell membrane, CAPs exhibit a membranolytic effect through different modes, such as bilayer disruption, carpeting, toroidal pore formation, and barrel-stave formation [48]. Furthermore, unlike traditional chemotherapeutics, the electrostatic interaction-based specificity also confers CAPs the ability to kill not only the actively dividing but also the “dormant” cancer cells [48]. In addition, the anti-cancer effect of CAPs is also associated with various membrane-dependent mechanisms, such as activating apoptotic pathways via DNA fragmentation and cytochrome *c* release, inducing mitochondrial membrane permeabilization, inducing necrotic cell death via l-type calcium channel activation, ROS production, and activation of the MAPK/ERK signaling pathway, and inducing membrane blebbing and lysis [25,48,54,55].

NRC-03, a peptide derived from winter flounder, has recently been reported to cause cell membrane damage and selective death of cancer cells including breast cancer, multiple myeloma, and leukemia [[18], [19], [20]]. NRC-03 damages the cancer cell membrane enters the cytoplasm, interacts with mitochondria and DNA, and causes DNA fragmentation [18]. However, its efficacy and specificity to OSCC remain unclear. In this study, we showed that NRC-03 at a concentration of 15–75 μg/ml inhibited OSCC cell growth in dose and treatment duration manner. The higher concentration of NRC-03 (60 and 75 μg/ml) showed partial cytotoxicity toward normal HOK cells. NRC-03 at a concentration of 15–45 μg/ml inhibited HOK cell viability below 20%. NRC-03 at a concentration of 45 μg/ml was observed as a median lethal dose towards OSCC cells at 4 h treatment and cell viability was reduced by around 80% in 24 h treatment. These findings indicated the importance of the dose and duration of NRC-03 treatment for OSCC. We further investigated the mode of NRC-03-induced OSCC cell death. We found that NRC-03 dramatically upregulated apoptosis in OSCC cells as indicated by overexpression of annexin-V, DNA fragmentation, and increased caspase-3 activity. The inhibition of caspase activity rescued the NRC-03-induced DNA fragmentation and apoptosis in OSCC cells. Furthermore, intratumoral injection of NRC-03 robustly induced DNA fragmentation and inhibited OSCC tumor growth in mice ectopic model but did not show adverse effects on vital organs. Our results indicated that NRC-03 exerted a potent and specific anti-tumor effect on OSCC mainly via inducing the intrinsic/mitochondria-mediated apoptosis pathway in cancer cells.

Cellular uptake of NRC-03 was robustly higher and abundant in OSCC cells compared to HOK cells. This might be related to electrostatic interaction between the negatively charged cell membrane of cancer cells and positively charged NRC-03. The cell membrane blebbing is the morphological step required to generate apoptotic bodies [56], which causes the toroidal pore formation on the cell membrane, increases the membrane permeability, decreases the cell osmotic pressure, and induces cell apoptosis [57]. We observed that NRC-03 peptide bound to OSCC cell membrane, causing membrane blebbing. DNA is negatively charged and the surface charge of normal mitochondria is not electronegative. During the early stage of apoptosis, exposure to negatively charged cardiolipin in the outer membrane causes a negative surface charge in mitochondria [58,59]. Therefore, our results showed that NRC-03 entered cancer cells quickly and abundantly and targeted the nucleus and mitochondria of OSCC cells. These results indicated that the positive electrostatic property of NRC-03 could target negatively charged DNA and mitochondria in OSCC. On the other hand, during the process of intracellular trafficking to mitochondria, how NRC-03 escapes from proteolytic degradation in cytosol and lysosome remains largely unknown. In our study, we found that NRC-03 was rapidly targeted to, and showed significant co-localization with mitochondria. Furthermore, our data also showed that no significant co-localization of NRC-03 with lysosomes. Therefore, NRC-03 might be directly targeted to mitochondria without an esacape from lysosomes. And the rapidly internalized and accumulated NRC-03 with abundant amount may help to maximally escape from cytosolic proteolyzation.

Mitochondria are key organelles for several cellular functions, ranging from ATP production, metabolism, calcium homeostasis, and modulation of signaling events leading to cell survival or cell death [[60], [61], [62]]. mtROS is produced by the leakage of electrons at complexes I and III of the electron transport chain which leads to disruption of mitochondrial function [63,64]. mtROS is the main source of cellular ROS. We found that both cellular and mitochondrial oxidative stress were robustly upregulated through increasing oxygen consumption and expression of mitochondrial respiratory chain complex I in NRC-03-treated OSCC cells. Reports from the literature suggest a reciprocal link between mitochondrial shape change and mtROS generation [[64], [65], [66]]. During increased generation of mtROS, mitochondrial shape changes from tubular to donut or blob form [64]. Similarly, NRC-03-bound mitochondrial morphology was changed to a round and swollen structure. Since NRC-03 was colocalized in mitochondria and altered the mitochondrial shape, we further investigated the effect of NRC-03 on mitochondrial oxidative stress and mitochondrial function in OSCC cells. NRC-03 dramatically reduced mitochondrial membrane potential and ATP production. These effects of NRC-03 were alleviated by the treatment of non-specific antioxidant (NAC) or mitochondria-specific antioxidant MitoQ. Moreover, both antioxidants alleviated the cytotoxic and apoptotic effect of NRC-03 in OSCC cells. These results indicated that mitochondrial oxidative stress played a critical in NRC-03-induced aberrant mitochondrial shape, mitochondrial dysfunction, DNA damage, and apoptosis.

In the subsequent study, we took advantage of RNA-Seq to explore the mechanisms responsible for NRC-03-induced cell death. We found that NRC-03 regulated the differential expression of apoptosis, mitochondrial function, and mitochondrial oxidative stress-related genes. The pro-apoptotic factors *Chac1* and *Cdkn1a*, a subunit of the mitochondrial respiratory complex IV *Cox6b2*, and oxidative stress-related genes *Sesn2* and *Dusp1* were most significantly upregulated in NRC-03-treated OSCC cells*.* Cdkn1a is associated with p53/TP53-mediated inhibition of cellular proliferation in response to DNA damage. Cdkn1a regulates cell cycle progression, terminal differentiation, and apoptosis [[67], [68], [69]]. Chac1 catalyzes the cleavage of glutathione and is known as a pro-apoptotic component associated with oxidative stress and apoptosis pathways [[70], [71], [72]]. Sesn2 is a stress-inducible protein required for maintaining redox homeostasis [73] and is critically involved in cellular responses to various stresses. Sesn2 has a protective effect on physiological and pathological states mainly via regulating oxidative stress, endoplasmic reticulum stress, autophagy, metabolism, and inflammation [74]. Dusp1 dephosphorylates MAPK1/ERK2, regulates NF-κB activity, involves in the human cellular response to environmental stress, and promotes apoptosis [[75], [76], [77]]. In addition, GO and KEGG analysis further showed that NRC-03 induces CAL-27 cells' oxidative stress, apoptosis, and arrest cell cycle through regulation of MAPK, TNF, and NF-κB signaling pathways. MAPKs and NF-κB are involved in the regulation of diverse cellular processes such as cell survival and apoptosis, innate immune response, and stress responses to a variety of noxious stimuli [78]. ROS is one of the primary activators or amplifiers of cellular signaling pathways [79]. The activation of MAPK and NF-κB are the most classical signaling pathways for cell responses to ROS stimulation [80,81]. MAPK pathway comprises ERK, p38, and JNK signaling [82]. The MAPK/ERK pathway is a convergent signal node that receives input from many stimuli, including internal metabolic pressure and DNA damage pathways [83]. The genes related to the ERK1/2 cascade were significantly enriched in NRC-03-treated CAL-27 cells. These data altogether open the possibility that NRC-03 exposure induced oxidative stress, mitochondrial dysfunction, and apoptosis in OSCC cells via regulating the MAPK and NF-κB pathways.

The mRNA-Seq analysis showed that NRC-03 induced a significant upregulation *Ppif* gene that encodes CypD, a key regulator of mitochondrial mPTP in OSCC cells, which was also corroborated by RT-qPCR and Western blot analysis. mPTP is an important channel that controls material exchange and information transmission between mitochondria and cytoplasm and plays a key role in mediating apoptosis, necroptosis, and autophagy [[84], [85], [86]]. mPTP excessive opening leads to mitochondrial stress, which is characterized by impaired mitochondrial dysfunction, mitochondrial swelling, ROS generation, and release of cytochrome *c*, eventually leading to cell death [[87], [88], [89]]. Mitochondrial matrix-localized CypD is an initiator of mPTP opening and modulates the permeability of the mPTP in response to various stress stimuli [90,91]. CypD overexpression dysregulates its recruitment to the mPTP channel causing consecutive pore opening and profound swelling of the mitochondria [92]. Our results showed that either genetic knockout or pharmacological inhibition of CypD effectively rescued the NRC-03-induced mitochondrial dysfunction, apoptosis, and cell death. Taken together, our results indicated that NRC-03 induced apoptosis in OSCC cells via CypD-mPTP axis-mediated mitochondrial oxidative stress.

One limitation of this study is the adoption of an ectopic tumor model. The construction of an animal model of OSCC carcinoma in situ should be performed to further verify the anti-tumor effect of NRC-03 *in vivo*. Primary human OSCC tissues and cells can be used to provide substantial evidence for the clinical application of NRC-03.

## 5 Conclusion

NRC-03 specifically and abundantly enters OSCC cells and locates in mitochondria and nucleus, causing membrane blebbing, mitochondria swelling, and DNA fragmentation. NRC-03 treatment increases oxygen consumption rate, causes ROS production in mitochondria via respiratory complexes I, and activates MAPK/ERK and NF-κB pathways in OSCC cells. NRC-03 significantly increases CypD expression and stimulates mPTP opening, subsequently causing the mitochondrial oxidative stress-mediated reduction in ATP production leading to apoptosis of OSCC cells. These findings suggest a promising application potential of NRC-03 to treat OSCC.

## Authorship

Study conception and design: GW, JLP, and TF. Cellular studies and data analysis: DH and FJH. The flow cytometry and immunofluorescence assays: DH, YXM, and LY. Western blot assay: YXM, ZCZ, and ATW. Animal experiments: DH, LY, and YHZ. Drafting of the manuscript: DH and JLP. Critical revision: GW and JLP. All authors approved the final version of the manuscript.

## Declaration of competing interest

The authors declare that the research was conducted in the absence of any commercial or financial relationships that could be construed as a potential conflict of interest.

## List of abbreviations

ANT: adenine nucleotide translocator
CAPs: cationic antimicrobial peptides
CLSM: confocal laser scanning microscope
CsA: cyclosporine A
CypD: cyclophilin D
DCFH-DA: 2′,7′-dichlorodihydrofluorescein diacetate
DMEM/F-12: Dulbecco's Modified Eagle Medium/Nutrient Mixture F-12
H&E: hematoxylin and eosin
HOK: human oral keratinocytes
H_2_O_2_: hydrogen peroxide
MitoQ: mitoquinone
mPTP: mitochondrial permeability transition pore
mtROS: mitochondrial reactive oxygen species
MW: molecular weight
NAC: *N*-acetyl-l-cysteine
NOX: NADPH oxidases
OCR: oxygen consumption rate
OSCC: oral squamous cell carcinoma
p*I*: isoelectric point
PMSF: phenylmethanesulfonyl fluoride
PPIF: peptidylprolyl isomerase F
PVDF: polyvinylidene difluoride
ROS: reactive oxygen species
TRITC: tetramethylrhodamine
TUNEL: terminal deoxynucleotidyl transferase dUTP nick-end labeling
