# Supplementary material for: Cationic antimicrobial peptide NRC-03 induces oral squamous cell carcinoma cell apoptosis via CypD-mPTP axis-mediated mitochondrial oxidative stress
Source: Redox Biol. 2022 May 28;54:102355. doi: 10.1016/j.redox.2022.102355 (PMC9511698; doi:10.1016/j.redox.2022.102355)
Supplement: Multimedia component 1 [file mmc3.docx]

**Cationic antimicrobial peptide NRC-03 induces oral squamous cell carcinoma cell apoptosis via CypD-mPTP axis-mediated mitochondrial oxidative stress**

[Dan Hou](https://www.frontiersin.org/people/u/1016466)^1,2#^, Fengjun Hu^3#^, Yixin Mao^4,5,6#^, Liang Yan^7^, Yuhui Zhang^1^, Zhichao Zheng^1^, Antong Wu^1^, [Tymour Forouzanfar](https://www.frontiersin.org/people/u/1240688)^2^, Janak L. Pathak^1^*, Gang Wu^2,8^*

1. Affiliated Stomatology Hospital of Guangzhou Medical University, Guangdong Engineering Research Center of Oral Restoration and Reconstruction, Guangzhou Key Laboratory of Basic and Applied Research of Oral Regenerative Medicine, Guangzhou, Guangdong 510182, China;
2. Department of Oral and Maxillofacial Surgery/Oral Pathology, Amsterdam UMC/VUmc and Academic Centre for Dentistry Amsterdam (ACTA), Vrije Universiteit Amsterdam, Amsterdam Movement Science, Amsterdam, 1081 HZ Netherlands;
3. Institute of Information Technology, Zhejiang Shuren University, Hangzhou, Zhejiang, 310000 China;
4. Department of Prosthodontics, School and Hospital of Stomatology, Wenzhou Medical University, Wenzhou 325027, China;
5. Institute of Stomatology, School and Hospital of Stomatology, Wenzhou Medical University, Wenzhou 325027, China;
6. Laboratory for Myology, Department of Human Movement Sciences, Faculty of Behavioural and Movement Sciences, Vrije Universiteit Amsterdam, Amsterdam Movement Sciences, Amsterdam, 1081 HZ, Netherlands
7. Department of Medical Biochemistry and Molecular Biology, School of Medicine, Jinan University, Guangzhou 510632, China;
8. Department of Oral Cell Biology, Academic Centre of Dentistry Amsterdam (ACTA), University van Amsterdam and Vrije Universiteit Amsterdam, Amsterdam, 1081LA Netherlands.

^#^: These authors contributed evenly.

*: Corresponding authors:

Gang Wu: Gustav Mahlerlaan 3004, 1081LA Amsterdam, the Netherlands. Email: g.wu@acta.nl.

Janak L. Pathak: 31 Huangsha Avenue, Guangzhou, Guangdong Province, 510140, P.R.China. Email: [j.pathak@gzhmu.edu.cn](mailto:j.pathak@gzhmu.edu.cn).

**
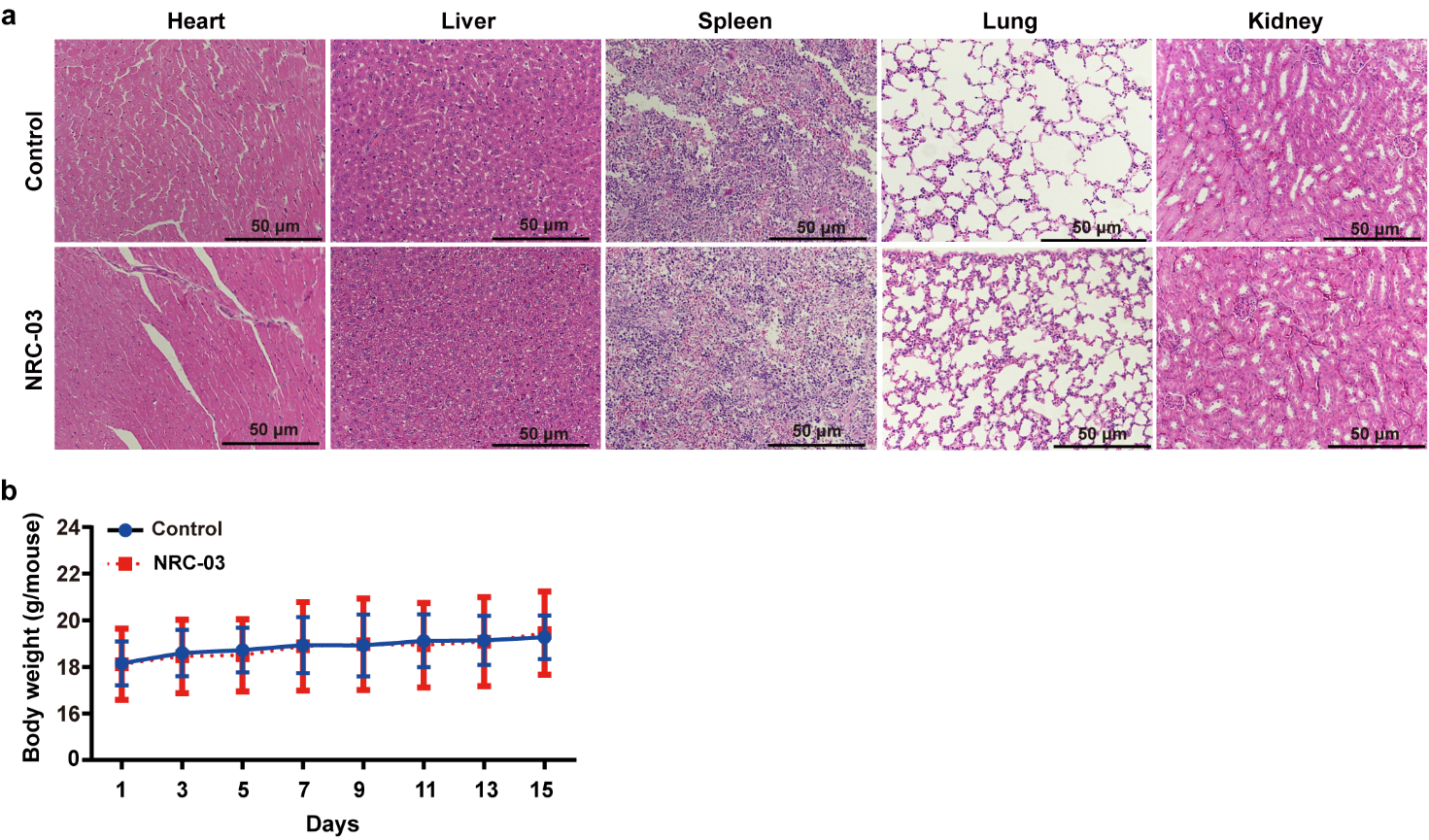
**

**Figure S1. NRC-03 did not cause observable tissue damage and weight loss in the ectopic tumor model of OSCC.** CAL-27-derived xenografts were treated with 125 μg NRC-03 every other day for 15 days. **(a)** Representative images of H&E-stained vital organs’ tissue sections of mice. **(b)** Bodyweight at different time points (*n* = 6).


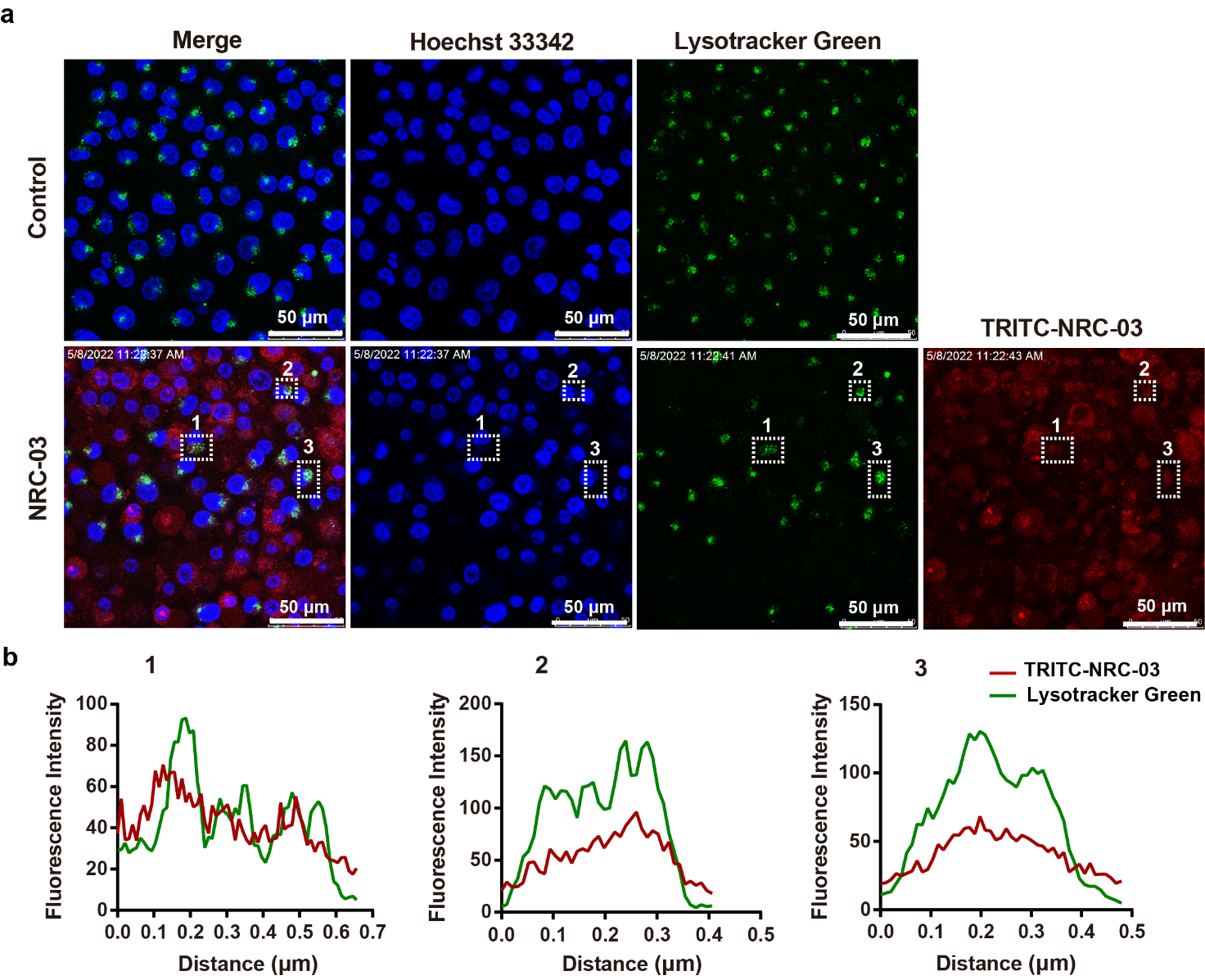


**Figure S2.** **NRC-03 didn’t bind to lysosomes in CAL-27 cells.** The nucleus, lysosomes, and peptides were visualized with CLSM by using Hoechst 33342 (Blue), Lysotracker Green (Green) and TRITC-NRC-03 (Red) respectively. **(a)** CLSM images of CAL-27 cells with or without NRC-03 treatment. **(b****)** Fluorescence signals analysis based on the white dotted frame in figure **a**.





**Figure S3. The changes in the fluorescence signal of the oxygen probe in CAL-27 cells with different treatments.** The CAL-27 cells were treated with different doses of NRC-03, and the fluorescence signal of the oxygen probe in cells was monitored over 2 h. Data are presented as mean, *n* = 5.

**
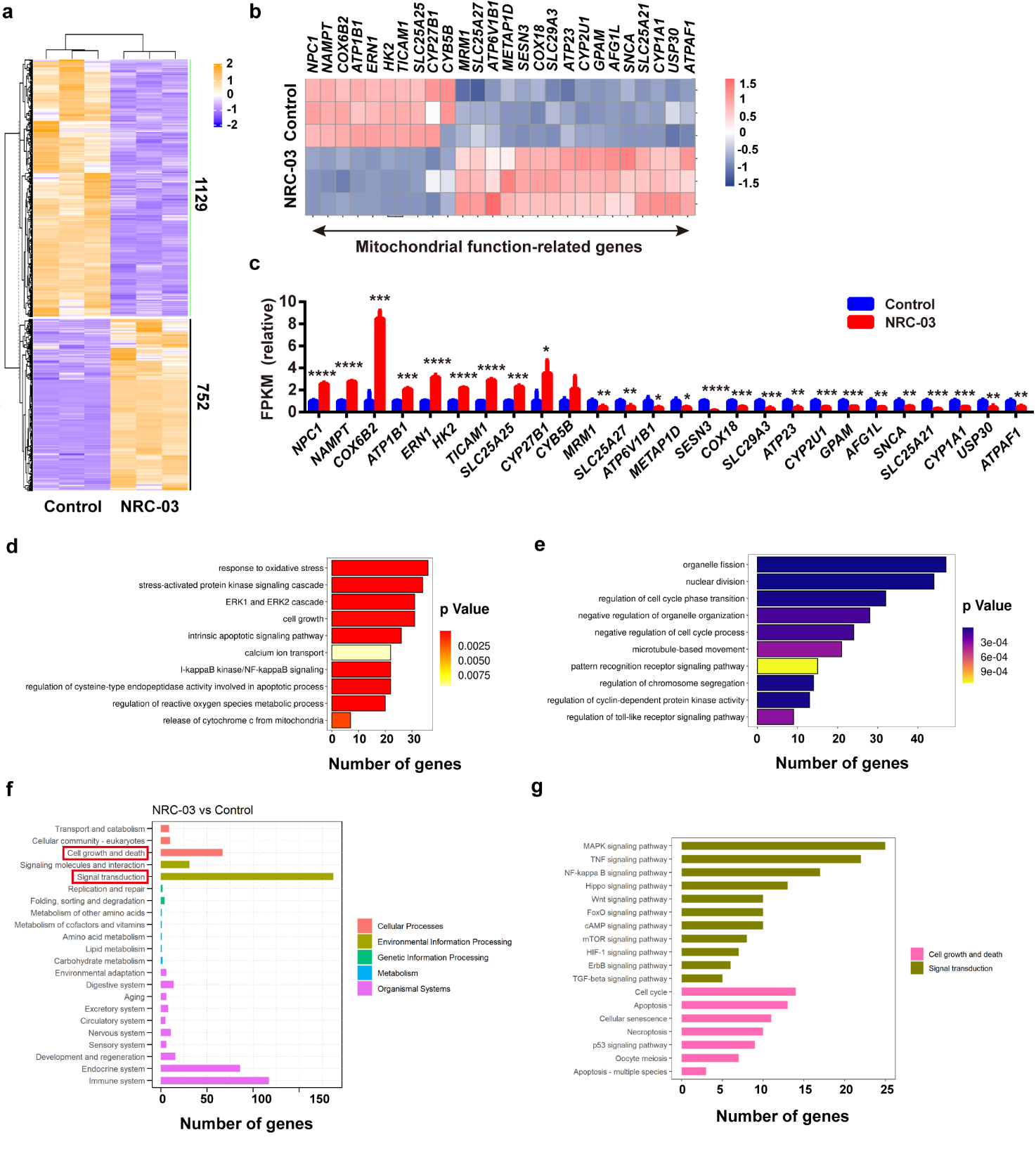
**

**Figure S4. RNA-seq analysis of effects of NRC-03 on CAL-27 cells.** **(a)** Heatmap of the differentially upregulated and downregulated genes. **(b)** Heatmap of differentially expressed mitochondrial function-related genes analyzed by RNA-seq. **(c)** Quantification of differentially expressed mitochondrial function-related genes analyzed based on FPKM. **(d)** GO pathway enrichment analysis of NRC-03-induced differentially upregulated genes. **(e)** GO pathway enrichment analysis of NRC-03-induced differentially downregulated genes. **(f)** KEGG pathway classification analysis. **(g)** The signaling pathways involved in signal transduction and cell growth and death. Data are presented as mean ± SD, *n* = 3. Significant difference compared with the respective control group, **p*<0.05, ***p*<0.01, ****p*<0.001, and *****p*<0.0001.

**

**

**Figure S5. CypD knockdown efficiency analyzed by RT-qPCR.** Data are presented as mean ± SD, *n* = 6. Significant difference between the respective group, *****p*<0.0001.

**Table S1. The primers used for RT-qPCR in this study.**

| **Target gene** | **Forward primer (5'->3')** | **Reverse primer (5'->3')** |
| --- | --- | --- |
| DDIT4 | TGAGGATGAACACTTGTGTGC | CCAACTGGCTAGGCATCAGC |
| IL1B | ATGATGGCTTATTACAGTGGCAA | GTCGGAGATTCGTAGCTGGA |
| TNFAIP3 | TCCTCAGGCTTTGTATTTGAGC | TGTGTATCGGTGCATGGTTTTA |
| BBC3 | GCCAGATTTGTGAGACAAGAGG | CAGGCACCTAATTGGGCTC |
| IL1A | AGATGCCTGAGATACCCAAAACC | CCAAGCACACCCAGTAGTCT |
| PPP1R15A | ATGATGGCATGTATGGTGAGC | AACCTTGCAGTGTCCTTATCAG |
| TNF | GAGGCCAAGCCCTGGTATG | CGGGCCGATTGATCTCAGC |
| CHAC1 | GAACCCTGGTTACCTGGGC | CGCAGCAAGTATTCAAGGTTGT |
| CDKN1A | TGTCCGTCAGAACCCATGC | AAAGTCGAAGTTCCATCGCTC |
| PPARGC1 | TCTGAGTCTGTATGGAGTGACAT | CCAAGTCGTTCACATCTAGTTCA |
| ATP6V1E2 | AGCCCGAAATGACCTCATCTC | CGGCAGCGTACAATCATCACA |
| PPIF | GAAGGCAGATGTCGTCCCAAA | GGAAAGCGGCTTCCGTAGAT |
| SESN2 | CCTCTGGGCGAGTAGACAAC | GGAGCCTACCAGGTAAGAACA |
| TXNRD1 | ATATGGCAAGAAGGTGATGGTCC | GGGCTTGTCCTAACAAAGCTG |
| RCAN1 | GCTCCGCCAAATCCAGACAA | GCTGCGTGCAATTCATACTTTTC |
| PTGS2 | CTGGCGCTCAGCCATACAG | CGCACTTATACTGGTCAAATCCC |
| DUSP1 | ACCACCACCGTGTTCAACTTC | TGGGAGAGGTCGTAATGGGG |
| NOS2 | AGGGACAAGCCTACCCCTC | CTCATCTCCCGTCAGTTGGT |
| GTSE1 | CAGGGGACGTGAACATGGATG | ATGTCCAAAGGGTCCGAAGAA |
| PMAIP1 | ACCAAGCCGGATTTGCGATT | ACTTGCACTTGTTCCTCGTGG |
| TNFRSF10B | GCCCCACAACAAAAGAGGTC | AGGTCATTCCAGTGAGTGCTA |
| NFKB2 | ATGGAGAGTTGCTACAACCCA | CTGTTCCACGATCACCAGGTA |
| DDIT3 | GGAAACAGAGTGGTCATTCCC | CTGCTTGAGCCGTTCATTCTC |
| TNFRSF10A | GCGGGGAGGATTGAACCAC | CGACGACAAACTTGAAGGTCTT |
| CFLAR | TCAAGGAGCAGGGACAAGTTA | GACAATGGGCATAGGGTGTTATC |
| RHOB | ATCCCCGAGAAGTGGGTCC | CGAGGTAGTCGTAGGCTTGGA |
| TP53BP2 | AGCTTGATCGCCTCTATAAGGA | CCCTCAGCTCATTAACACGCT |
| LRRK2 | ATGAGTGGCAATGTCAGGTGT | AATGTAAGCCTATGGAGCAAACA |
| GPX8 | TACTTAGGGCTGAAGGAACTGC | GGCTCCGATTCTCCAAACTGA |
| ATP8B3 | CGCTCAGTACCCCTATGGTCT | GCTGGGACGATGTTGTCCTT |
| PRODH | CCCTGCTTCGGCACTACAG | GGGCCTGGTATTGCTTGTCC |
| NLRX1 | GGCCTTTATACGCCACCAC | TGGACCGTGTCTAGGGCAAA |
| CYP1B1 | AAGTTCTTGAGGCACTGCGAA | GGCCGGTACGTTCTCCAAAT |
| SOD2 | GGAAGCCATCAAACGTGACTT | CCCGTTCCTTATTGAAACCAAGC |
| GCH1 | GTGAGCATCACTTGGTTCCAT | GTAAGGCGCTCCTGAACTTGT |
| NCF2 | CCAGAAGCATTAACCGAGACAA | CCTCGAAGCTGAATCAAGGC |
| GAPDH | GGACCTGACCTGCCGTCTAG | GTAGCCCAGGATGCCCTTGA |
| MT-ND1 | CTAATAAGTGGCTCCTTT | TGGTCTCTGCTAGTGTGG |
| MT-ND3 | TACCACAACTCAACGGCT | TTATGGAGAAAGGGACGC |
| MT-ND5 | ATAATAGTTACAATCGGC | TGGTTTTGAGTAGTCCTC |
| MT-ND6 | CTTCTAAGCCTTCTCCTA | ACCAATCCTACCTCCATC |
| SDHA | CAGCATGTGTTACCAAGCTGT | GGTGTCGTAGAAATGCCACCT |
| UQCRQ | ATCCGCACGTCTTCACTAAAG | TGGATCTCTCGAACTCTTCAGTC |
| UQCRC1 | GGGGCACAAGTGCTATTGC | GTTGTCCAGCAGGCTAACC |
| UQCR10 | TACTCCCTGCTGTTCCGC | CCTCACCCCCTCGTTGAT |
| UQCR11 | TGGTCAAGAACTGGGTCC | TGTGAAGGGTTTGTGTAA |
| MT-CO1 | TACTACTAACAGACCGCA | CCATACCTATGTATCCAA |
| MT-CO2 | AGTCCTGTATGCCCTTTT | CGTTGACCTCGTCTGTTA |
| MT-CO3 | AACACGAGAAAGCACATA | AAAAGGCTCAGAAAAATC |
| COX4I1 | GCGGCCTTGCTCTCTTC | GGTGGAAATTGCTCGCTT |
| ATP5F1A | AACTGATTATTGGTGACCGACAG | GGCAACAGTGGATCTCTTTTGA |
| CYBB | ACCGGGTTTATGATATTCCACCT | GATTTCGACAGACTGGCAAGA |
| NOX5 | GGCTCAAGTCCTACCACTGGA | GAACCGTGTACCCAGCCAAT |
| DUOX1 | GCAGCGATTTGATGGGTGGTA | AGGTGGGGTTCTCCCAAGG |

**Supplementary Movie 1. NRC-03 entered CAL-27 cells.** Peptide and nucleus were visualized by using TRITC-NRC-03 (Red) and Hoechst 33342 (Blue), respectively.

**Supplementary Movie 2. NRC-03 entered HOK cells.** Peptide and nucleus were visualized by using TRITC-NRC-03 (Red) and Hoechst 33342 (Blue), respectively.
